# Supplementary figures and images for: Relationships between growth mindsets and math achievement across socioeconomic status in 74 countries: Evidence from PISA 2022
Source: PLoS One. 2025 Nov 21;20(11):e0337039. doi: 10.1371/journal.pone.0337039 (PMC12637945; doi:10.1371/journal.pone.0337039)

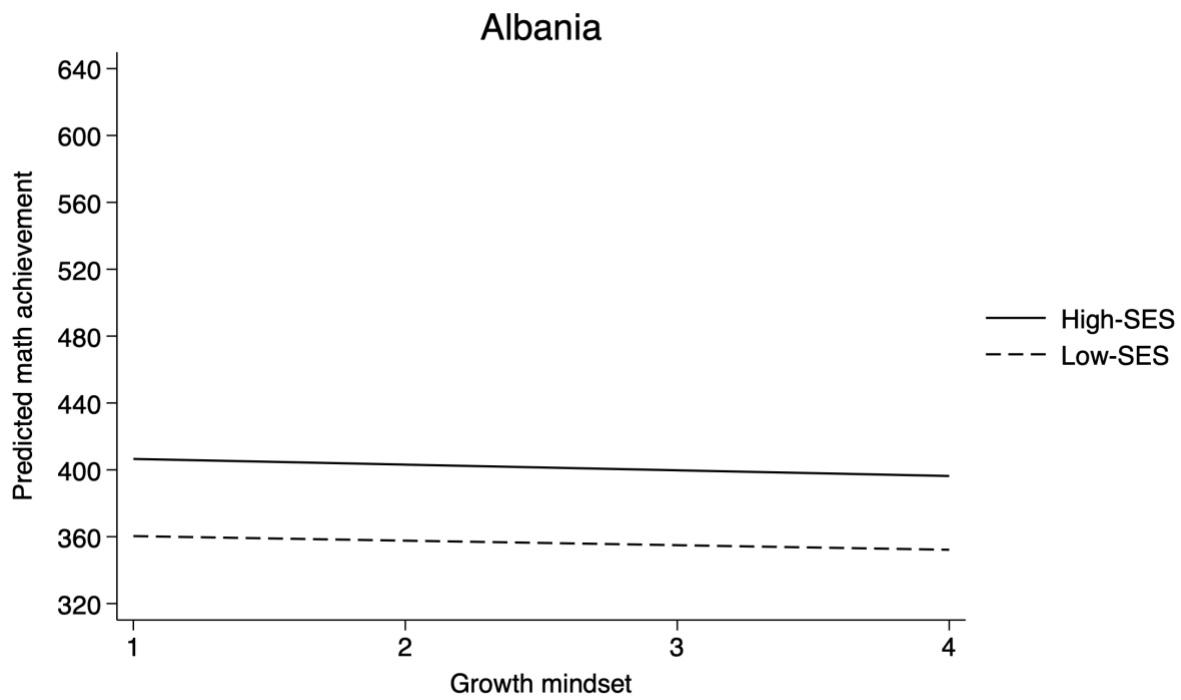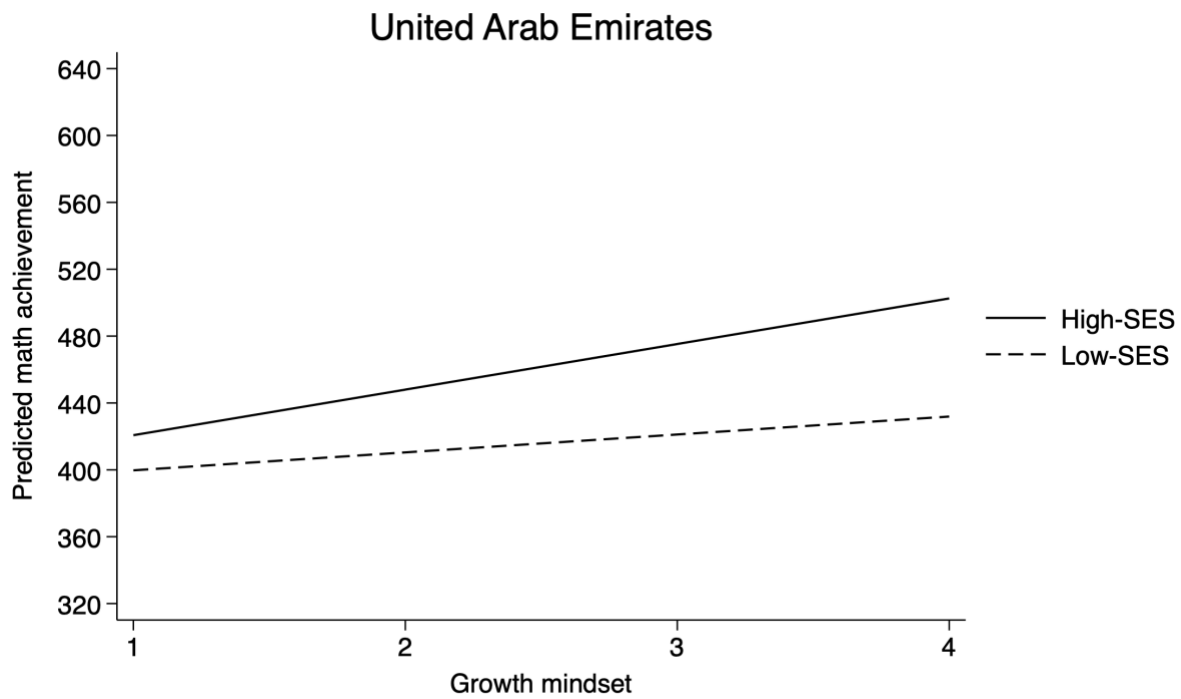

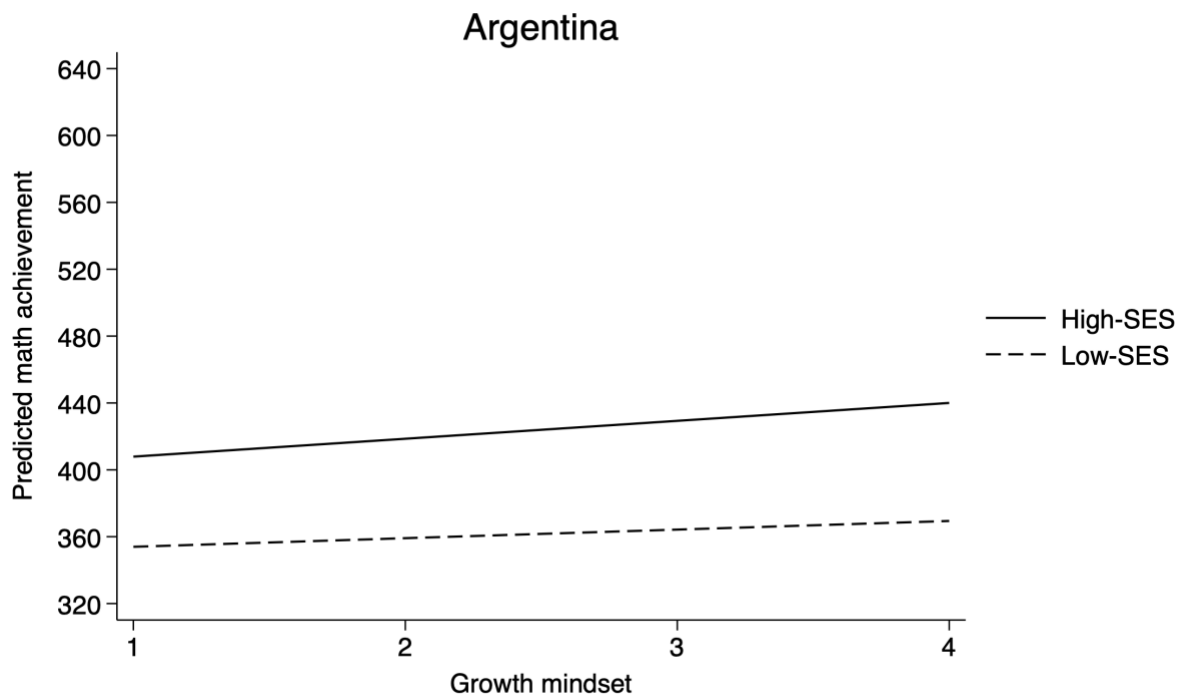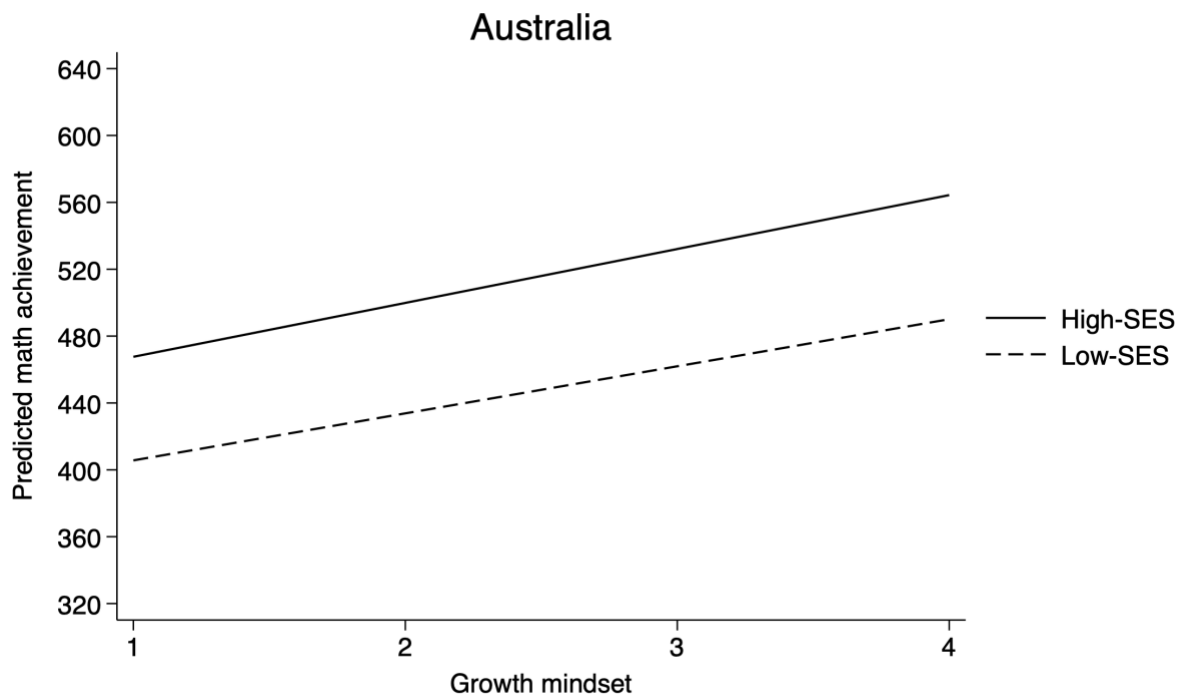

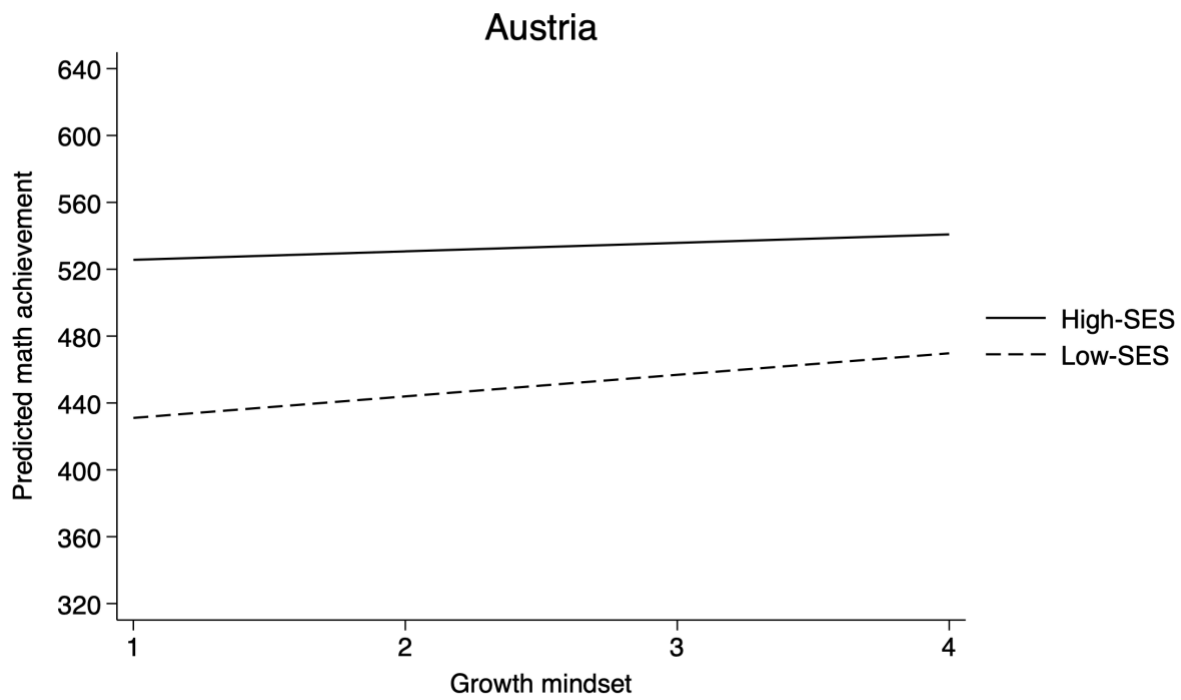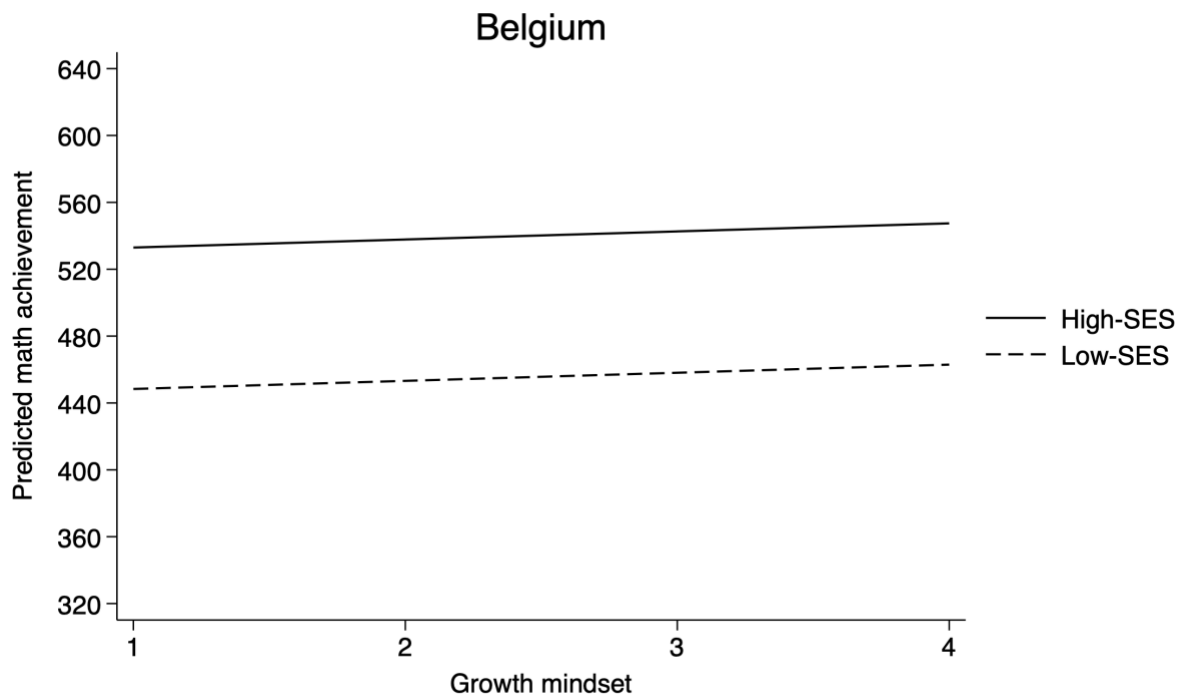

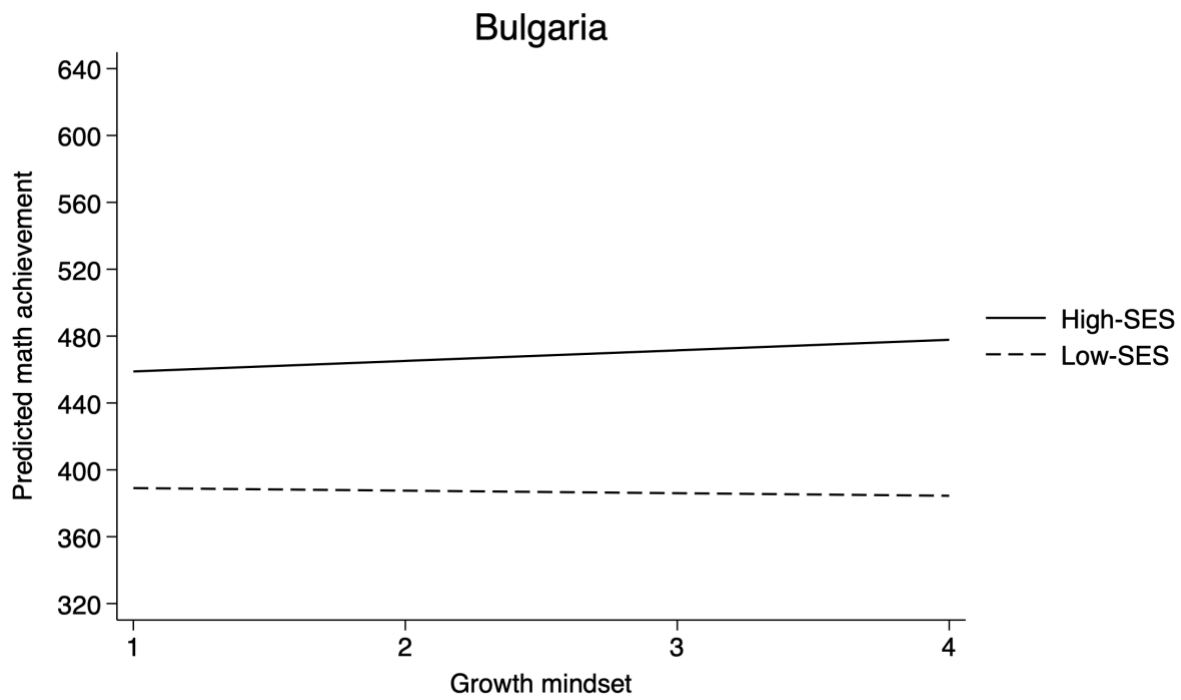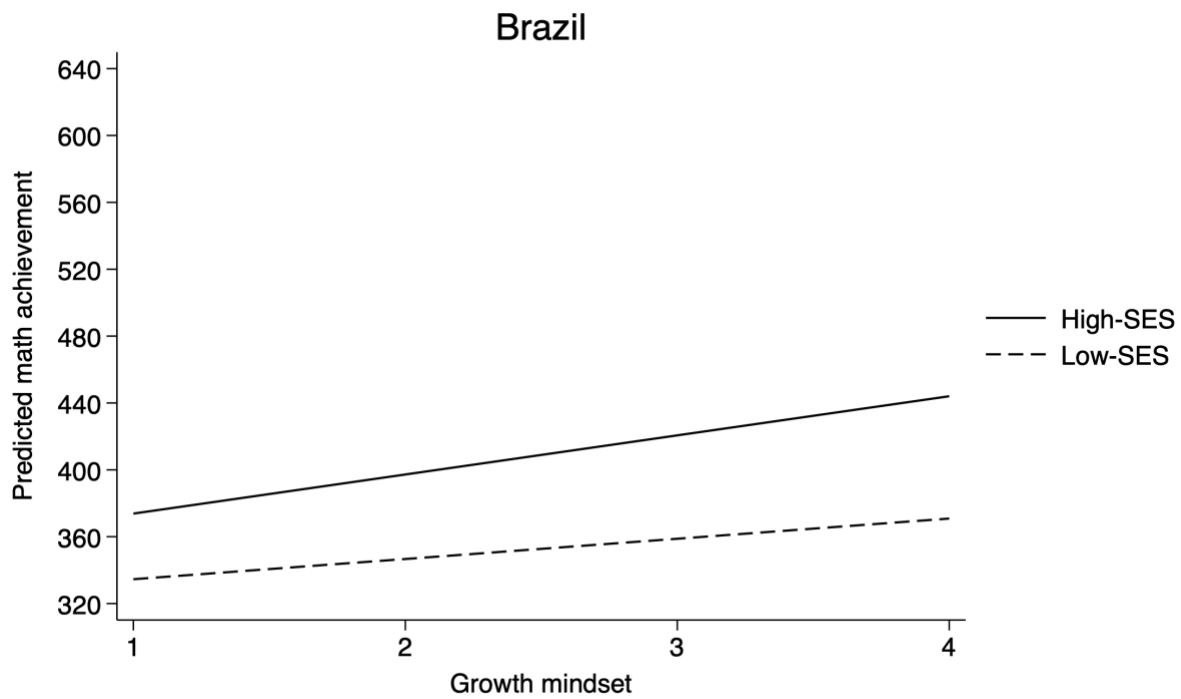

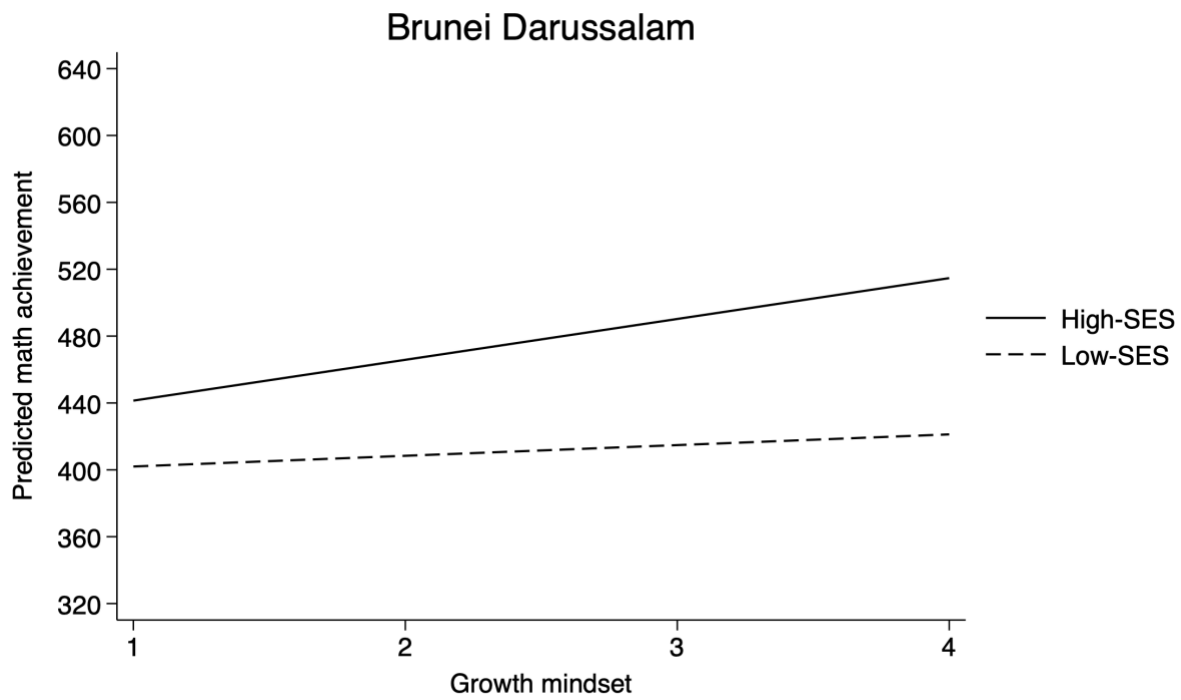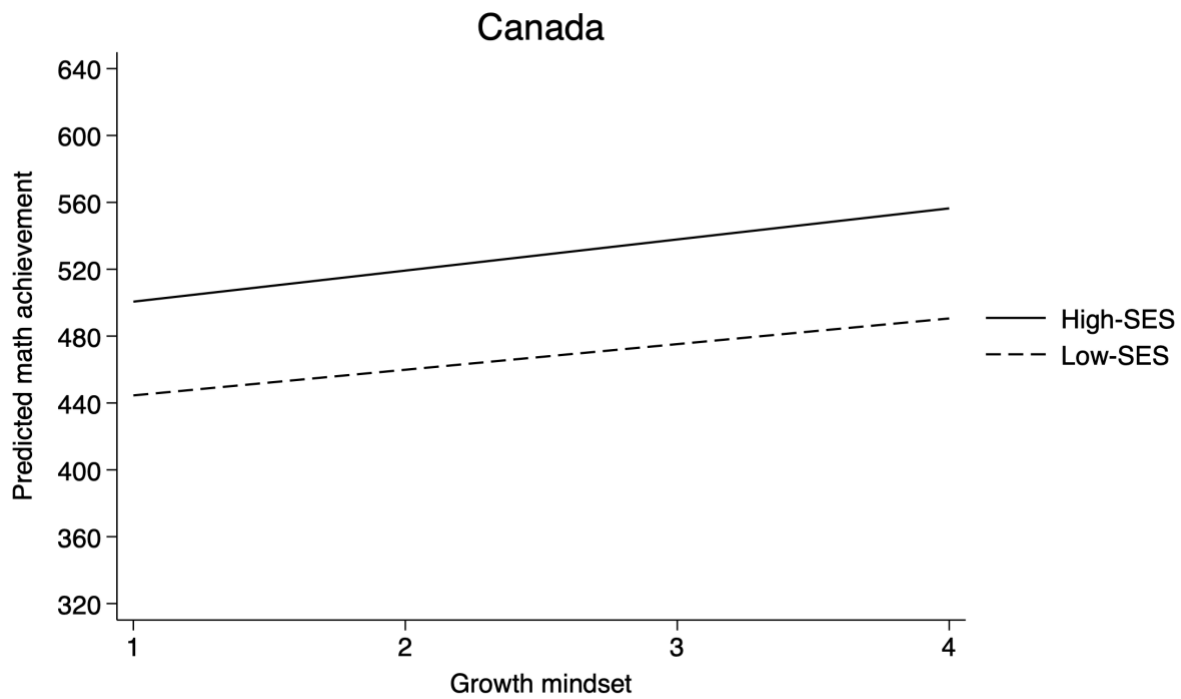

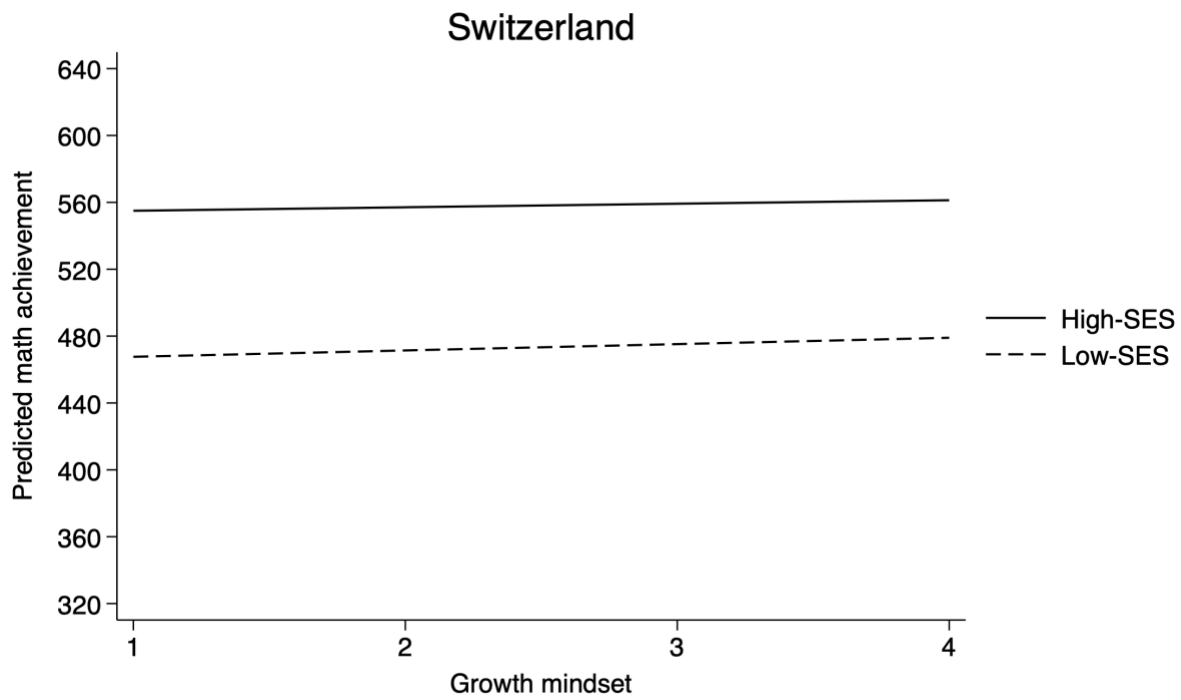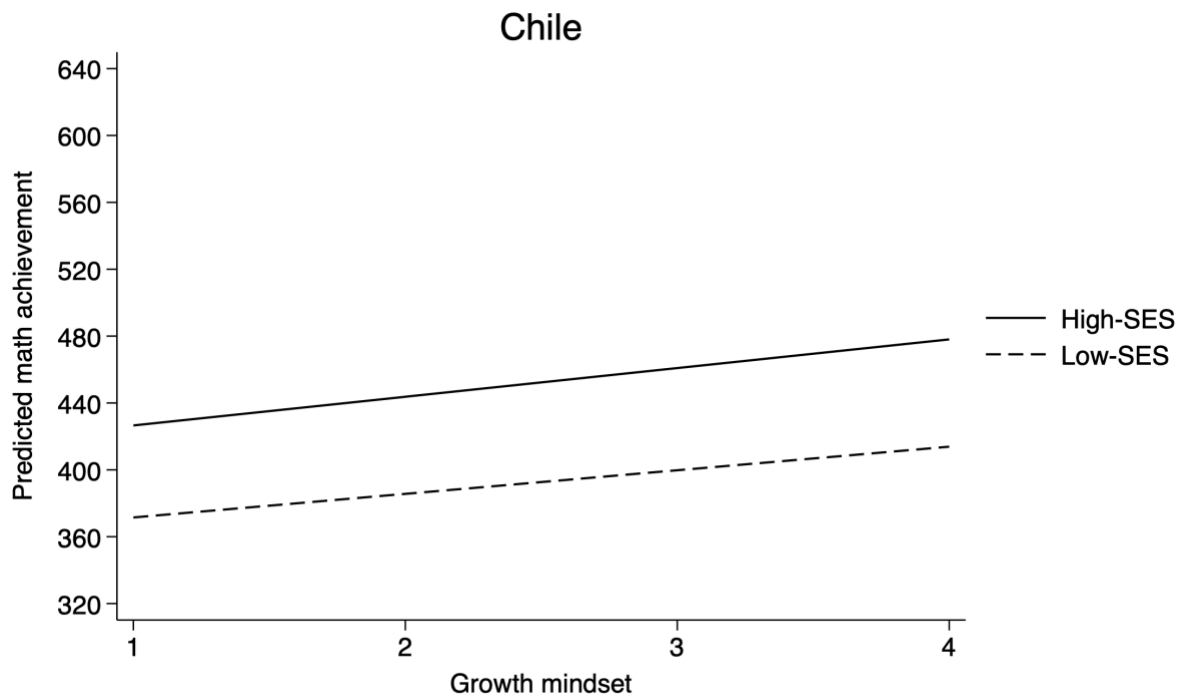

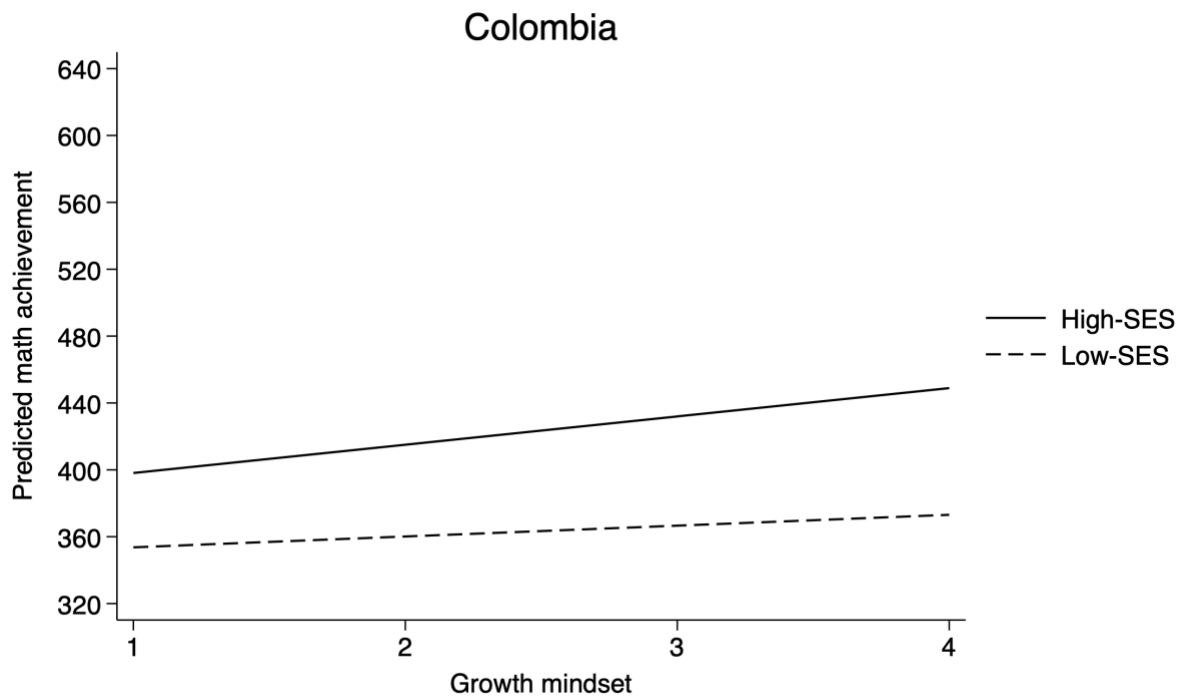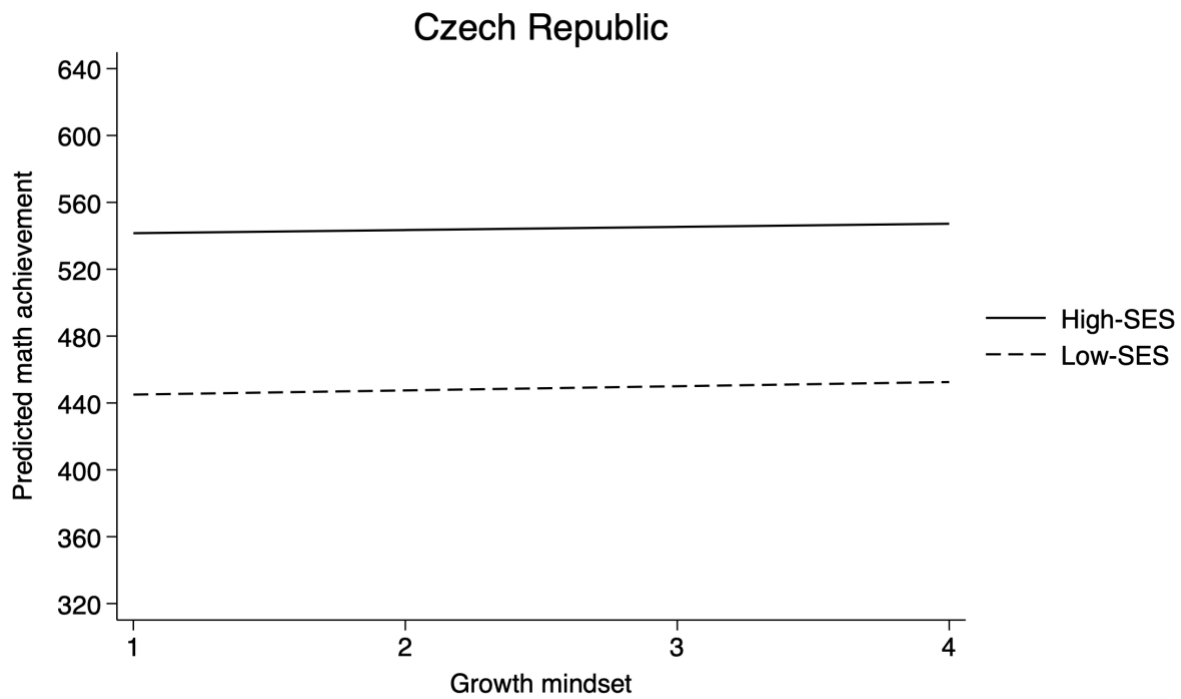

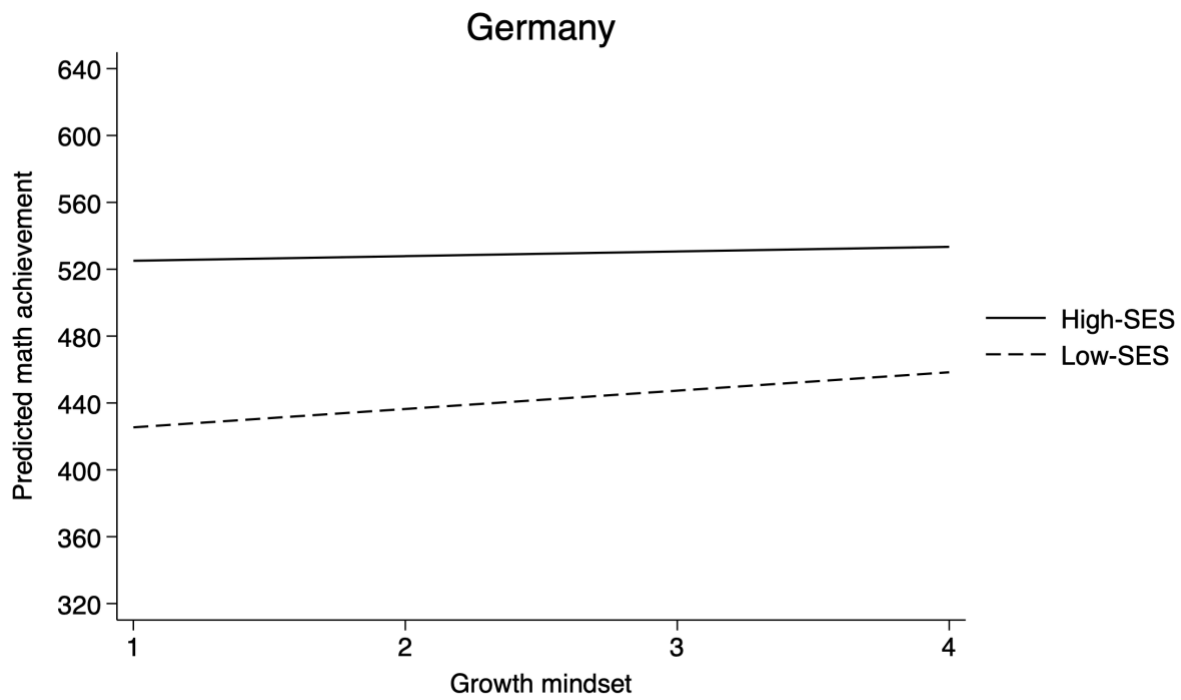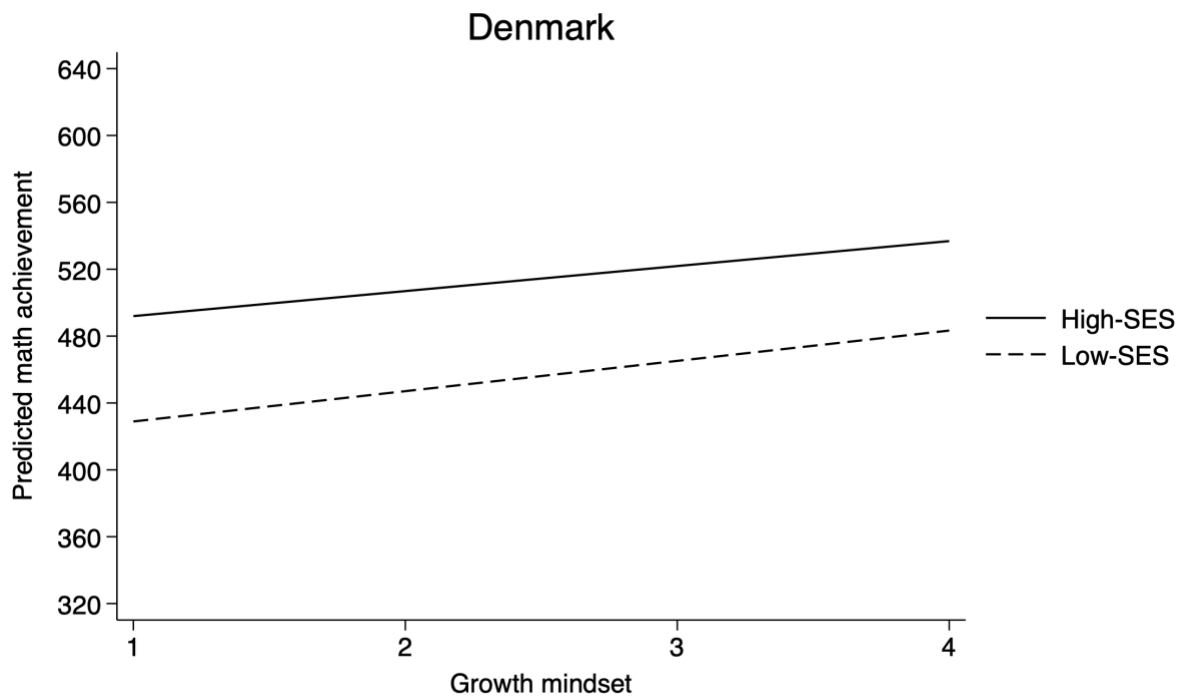

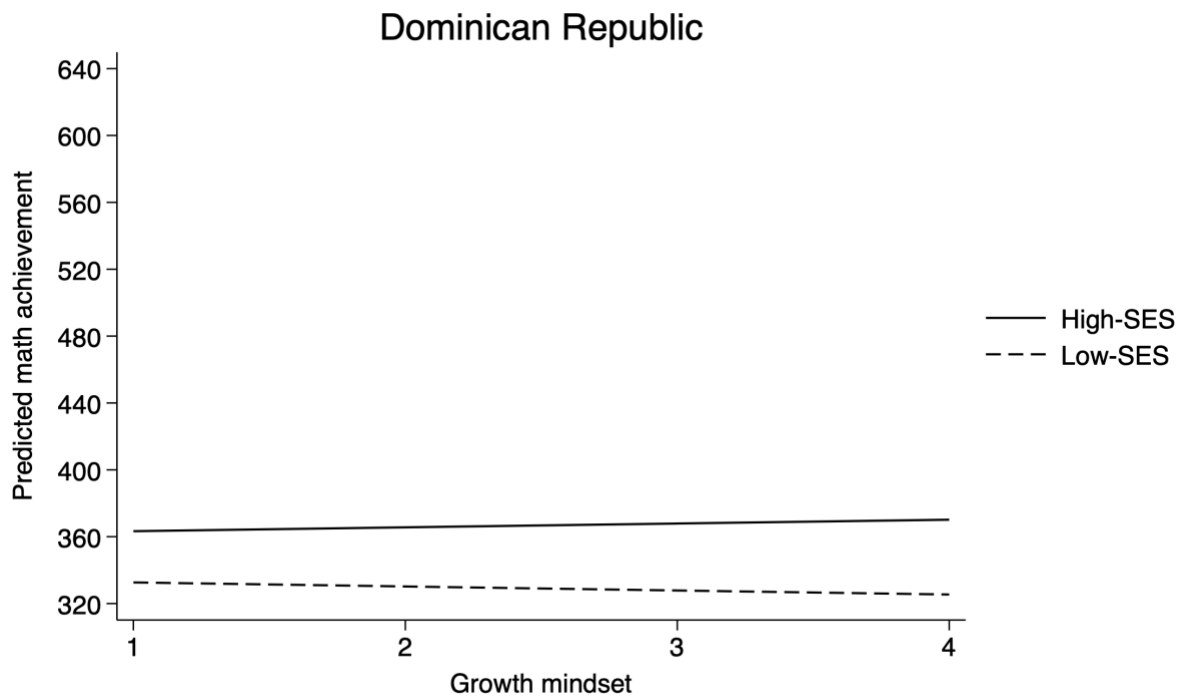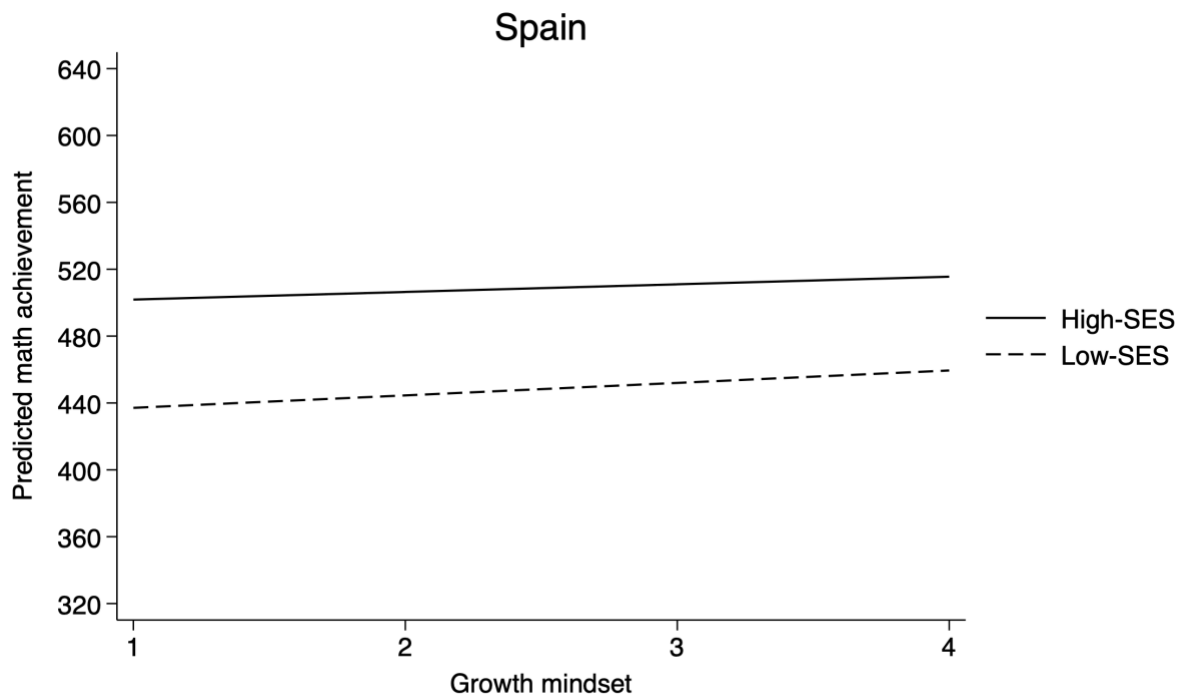

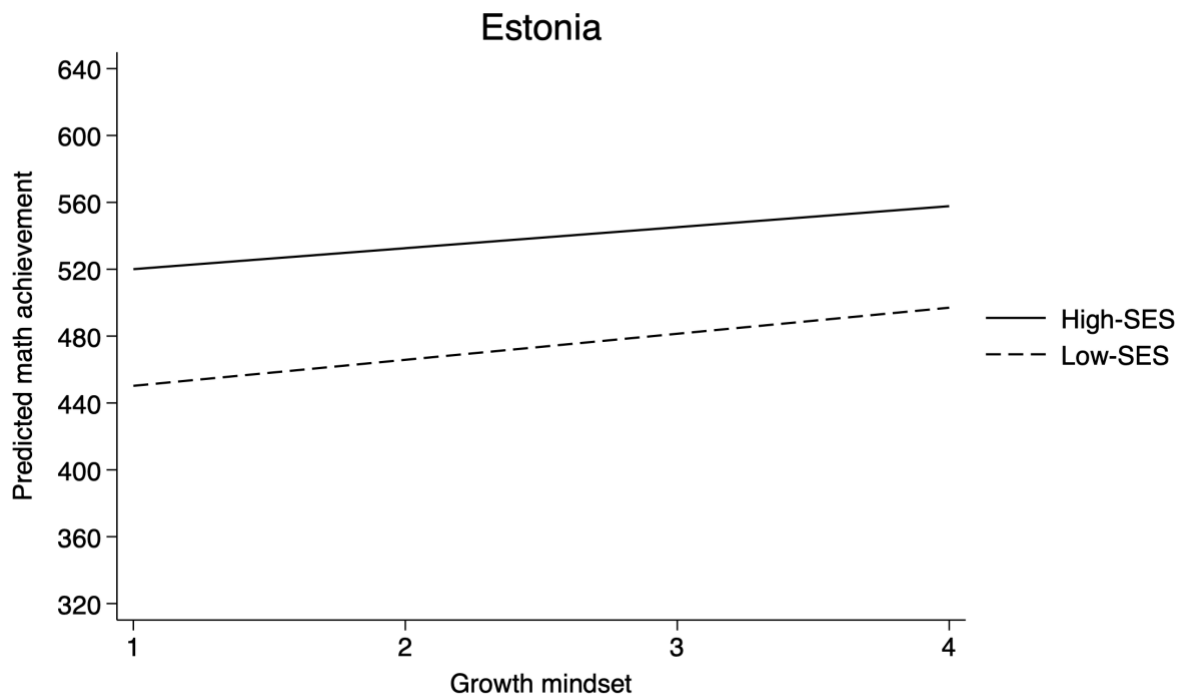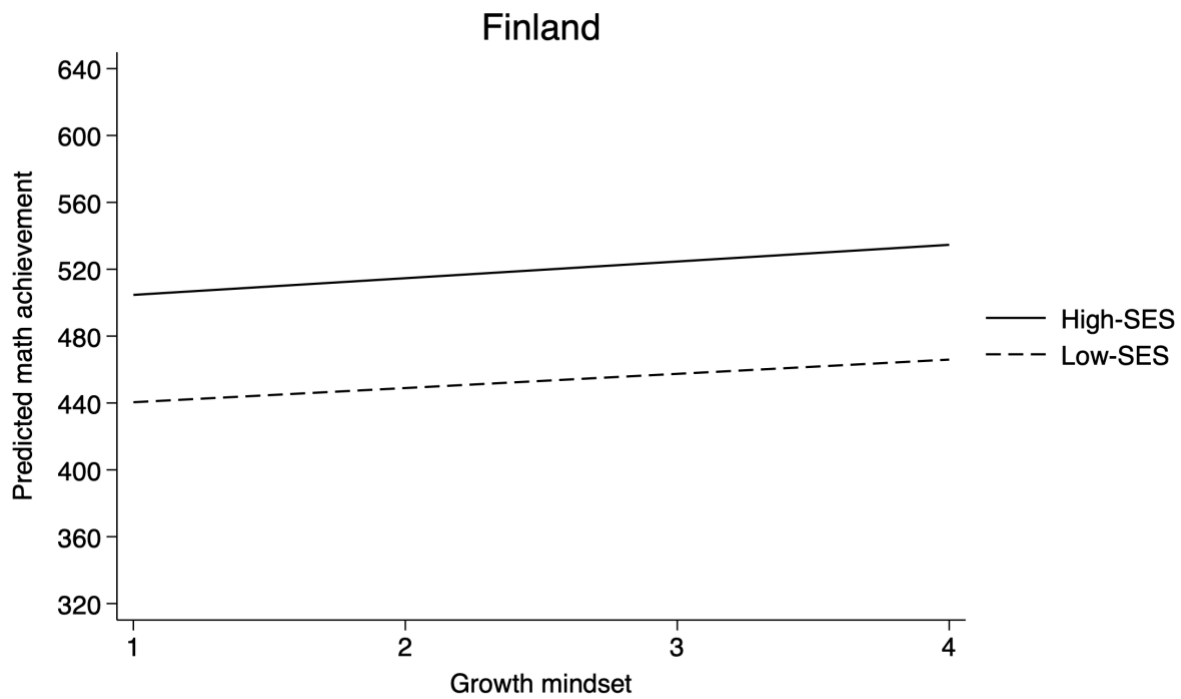

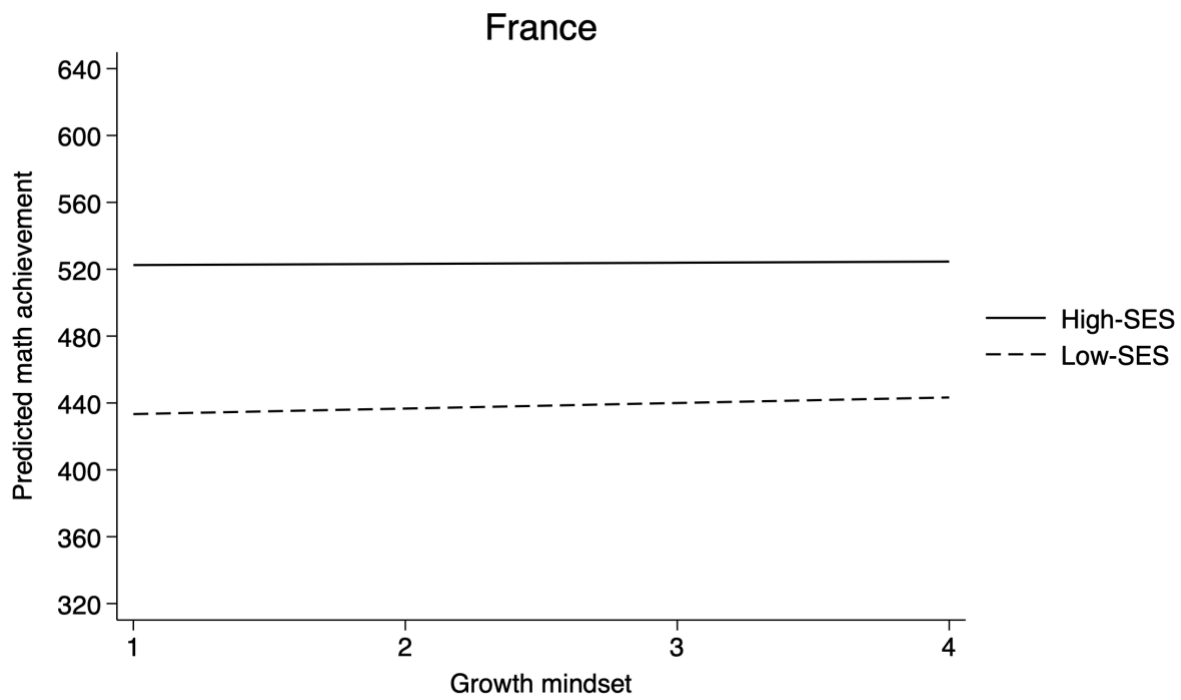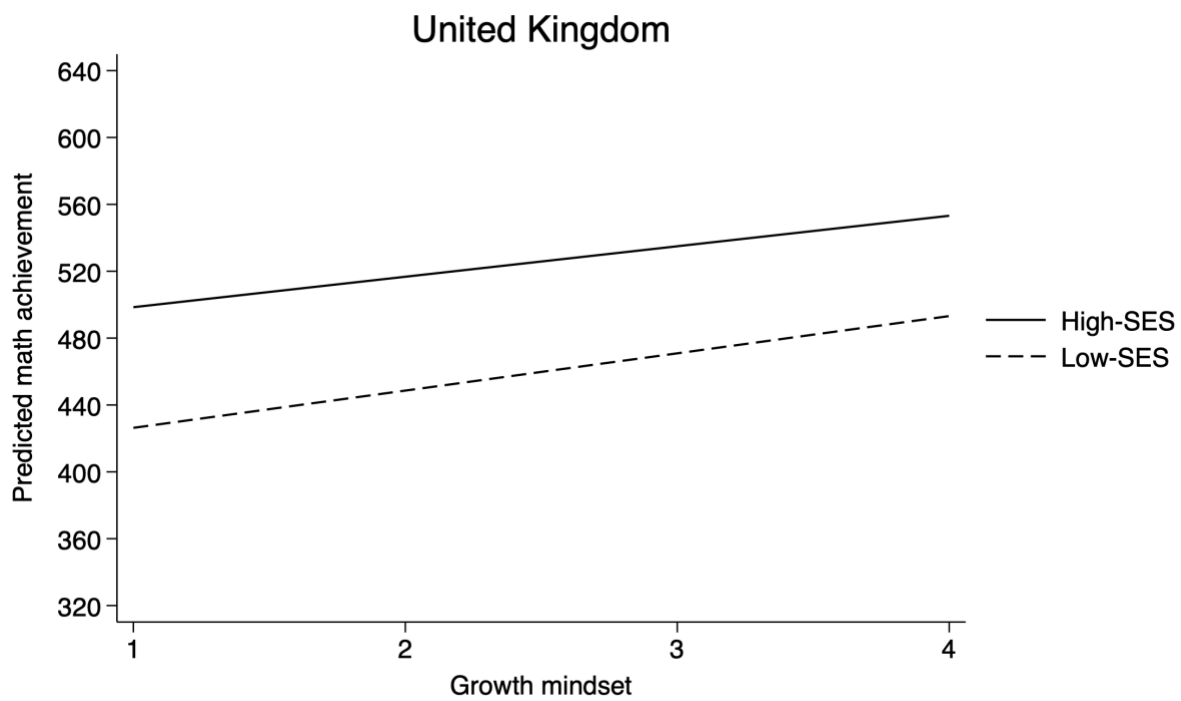

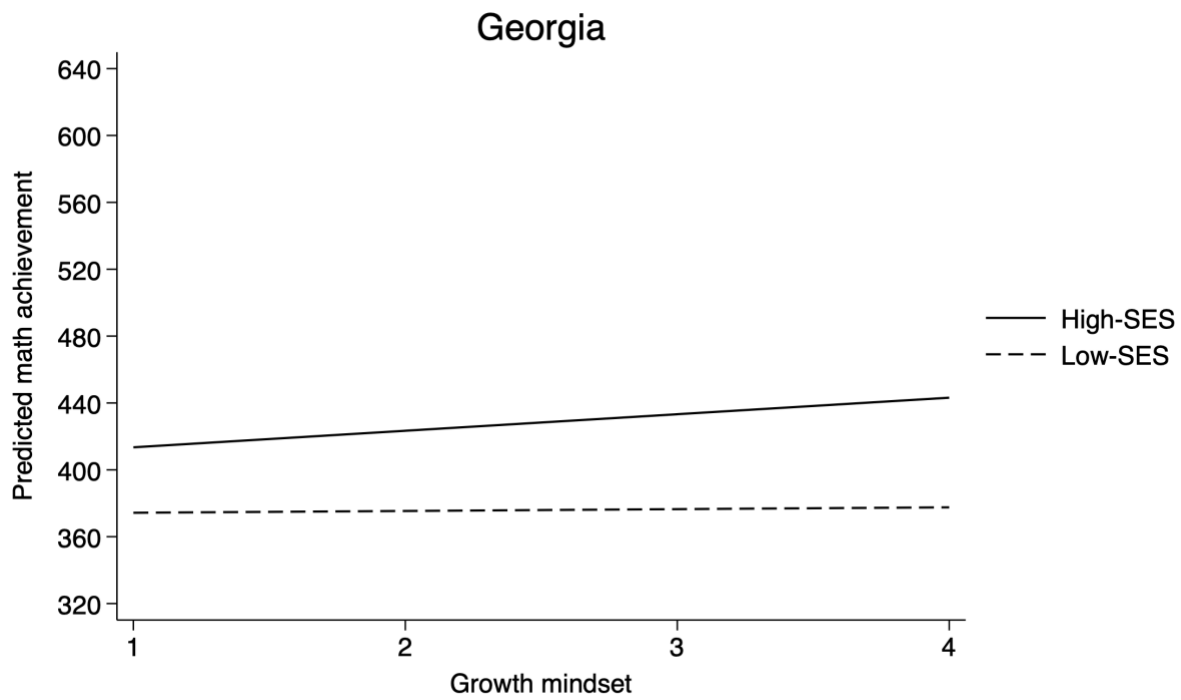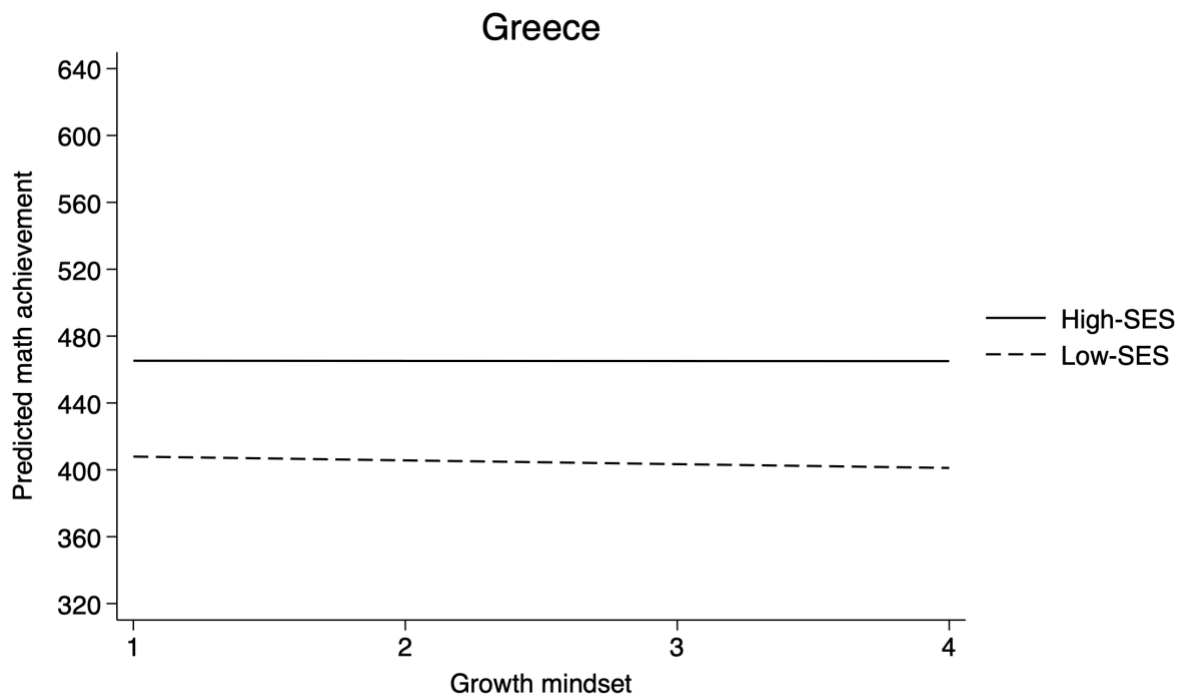

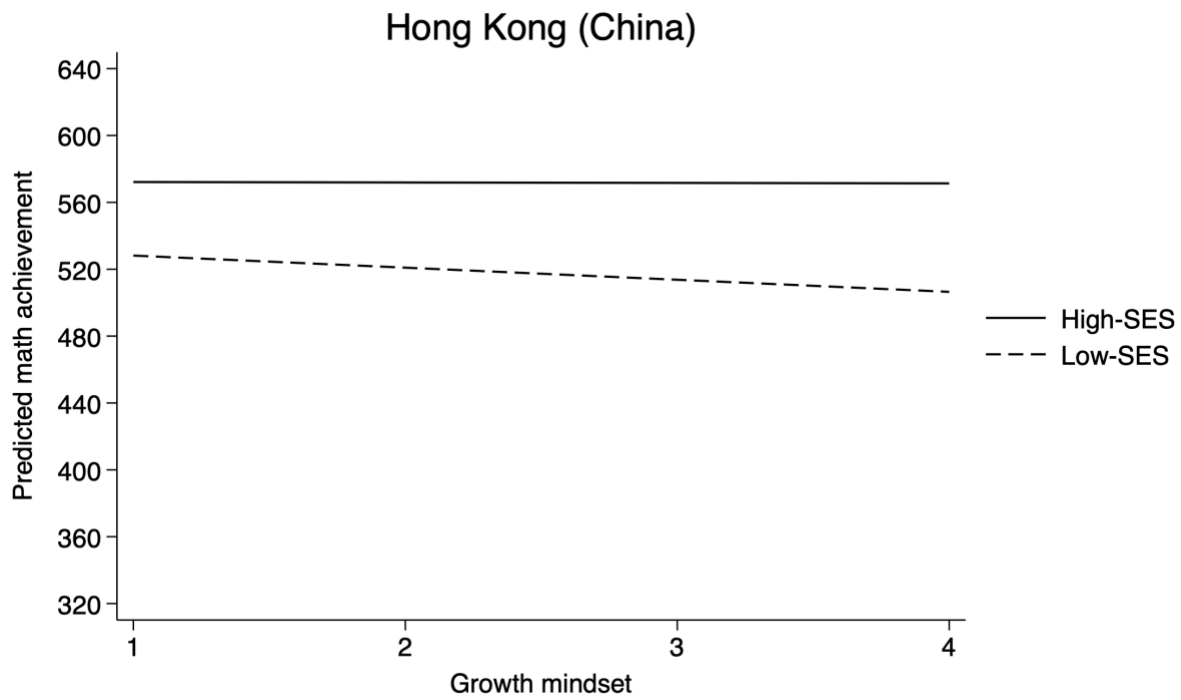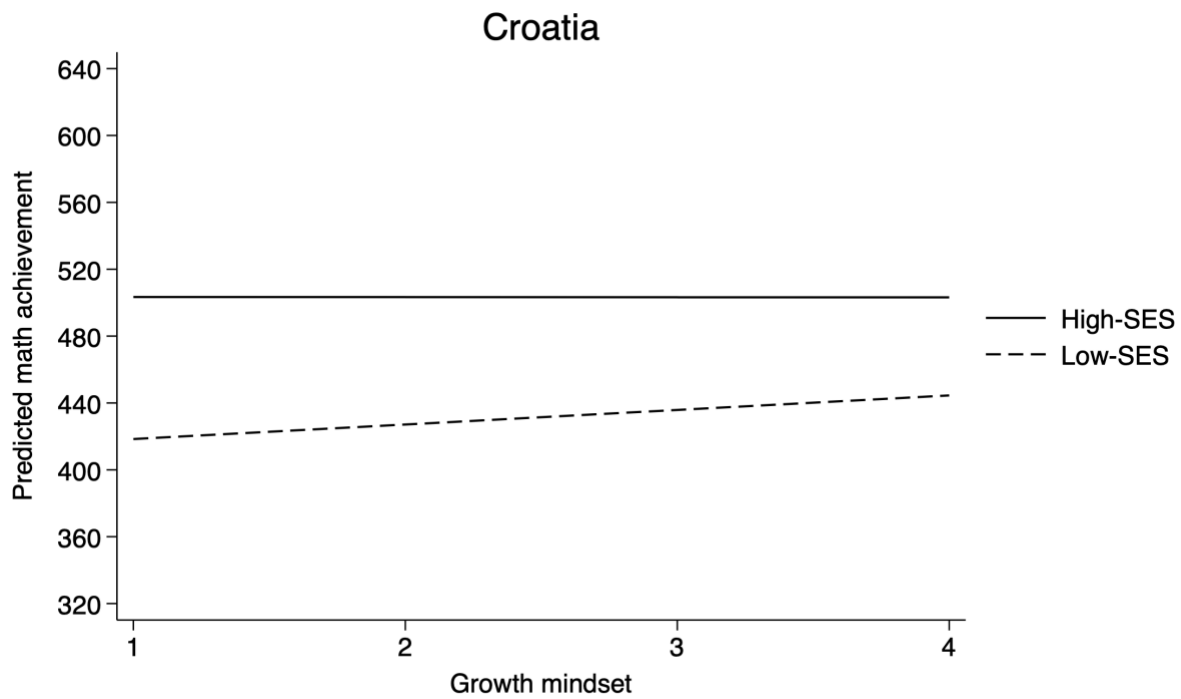

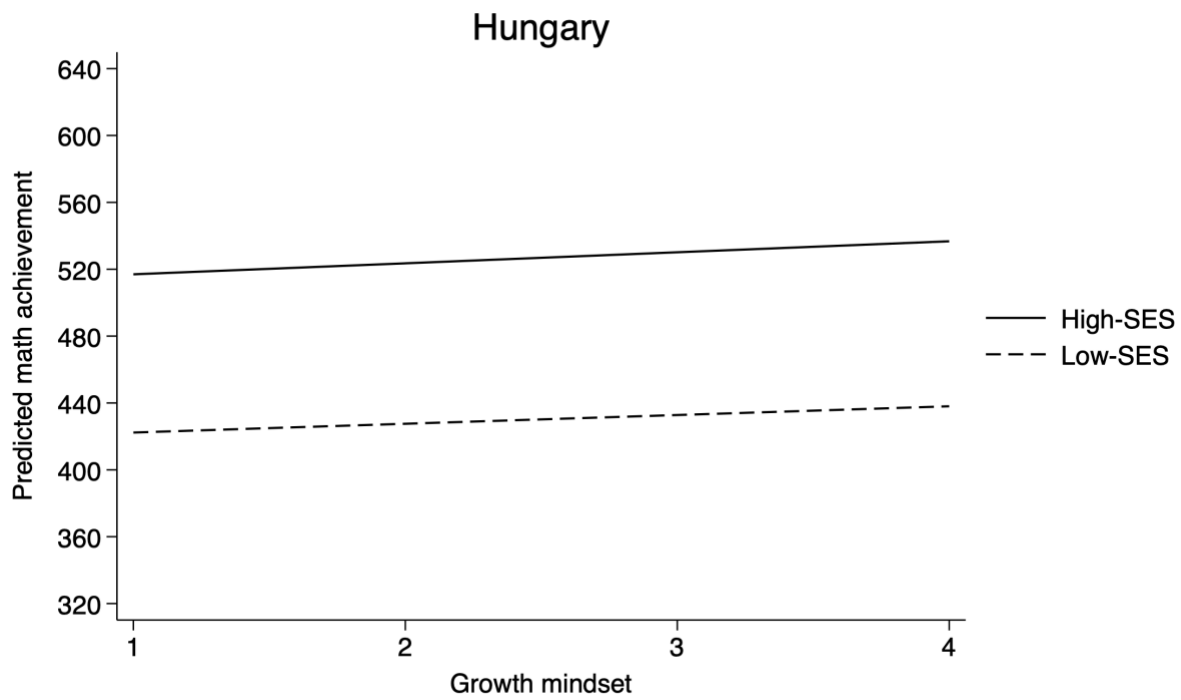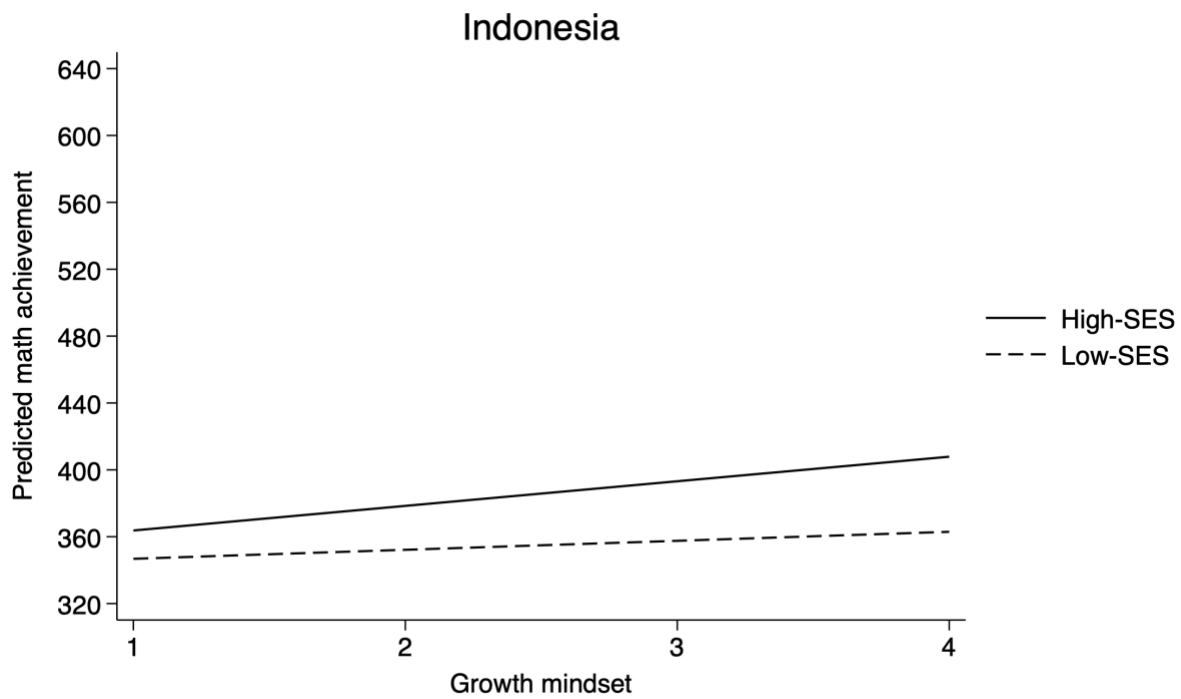

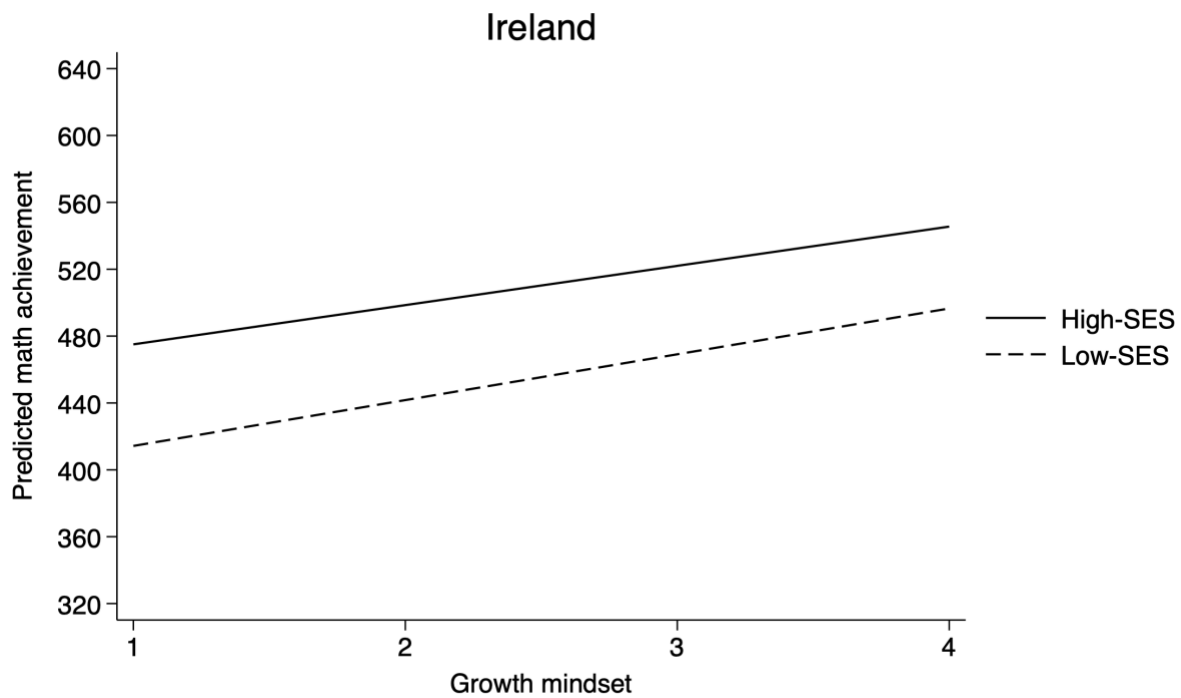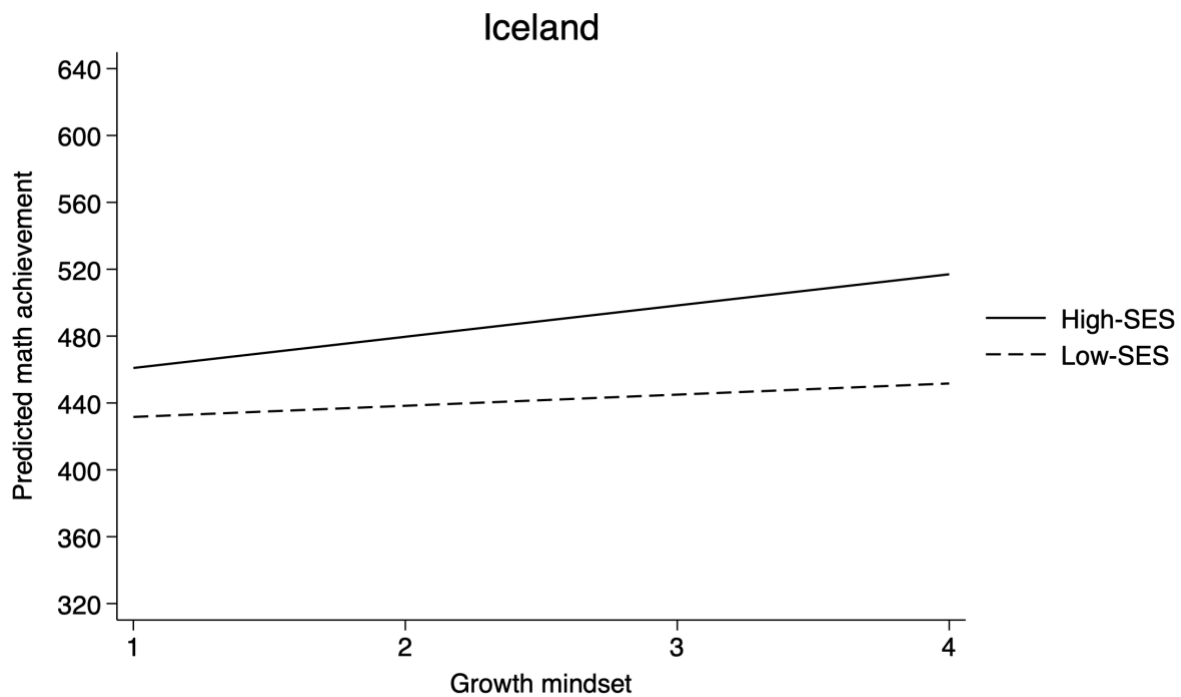

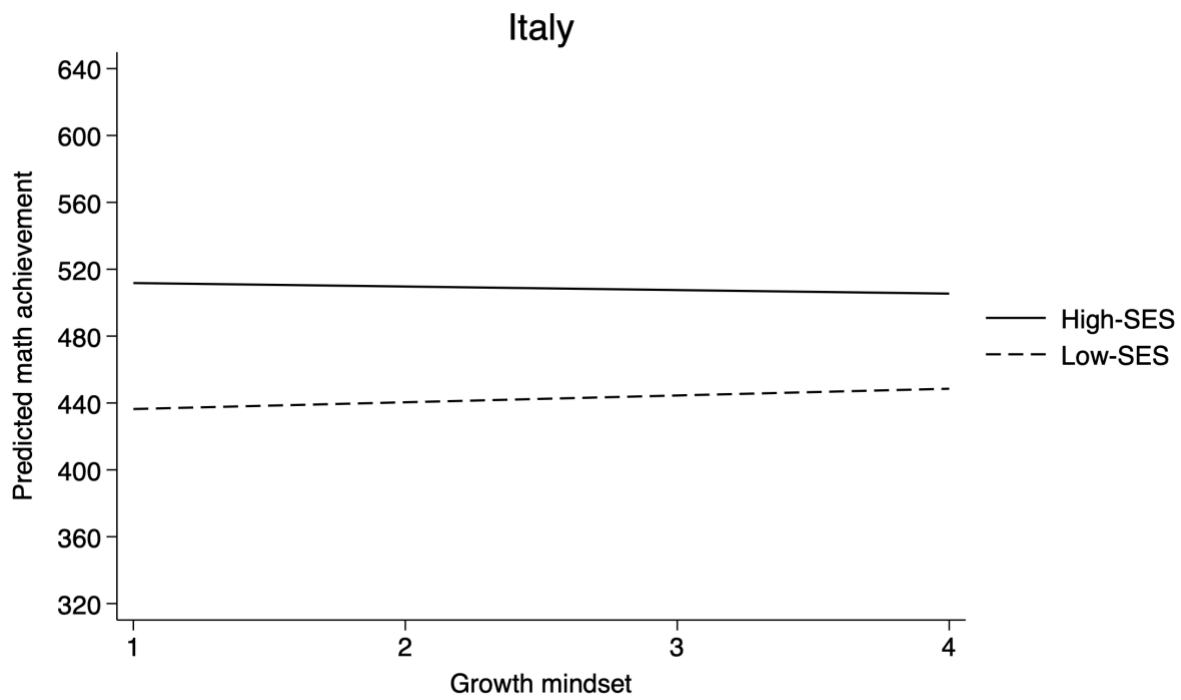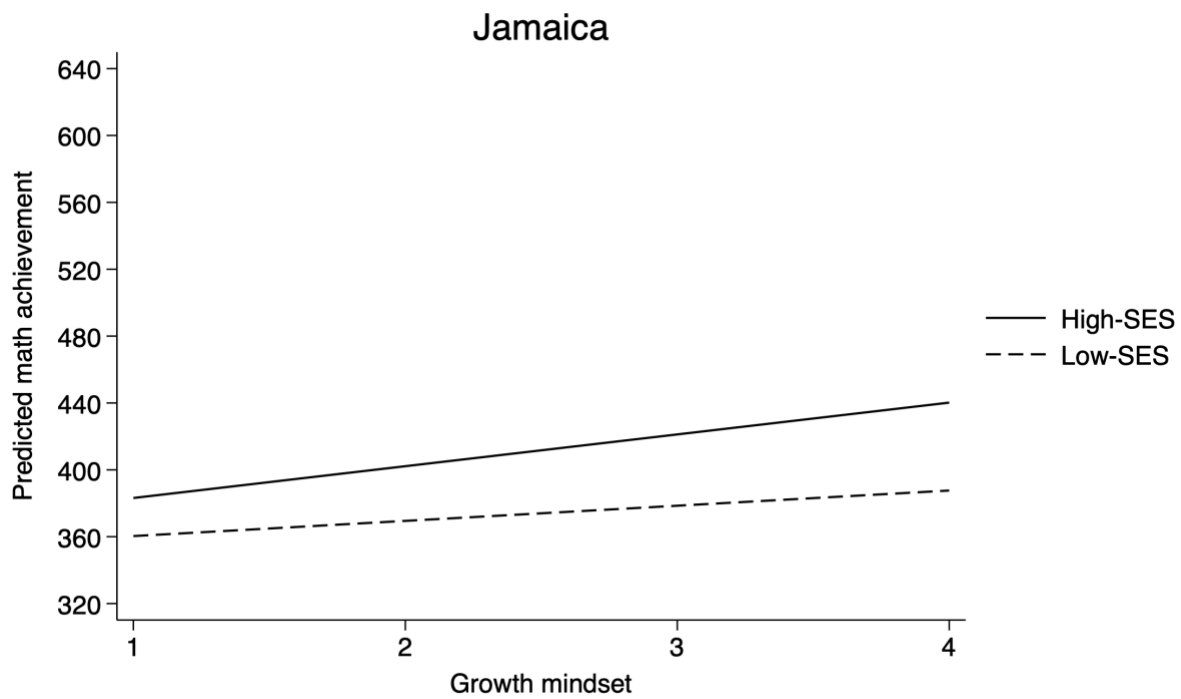

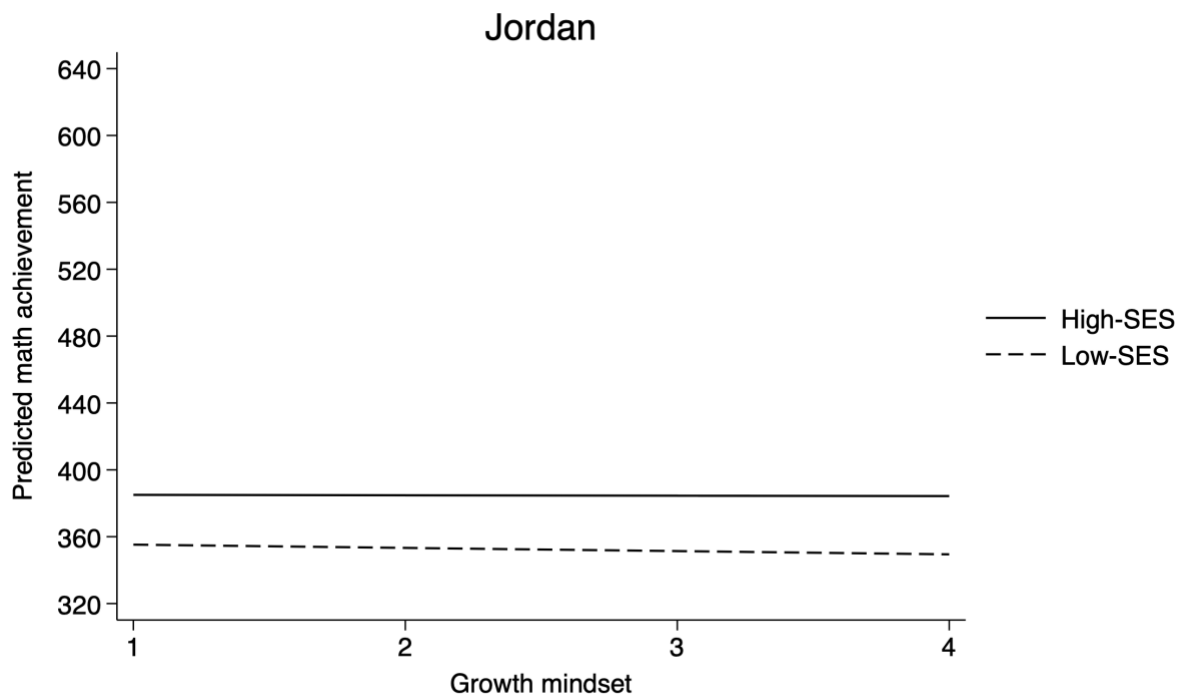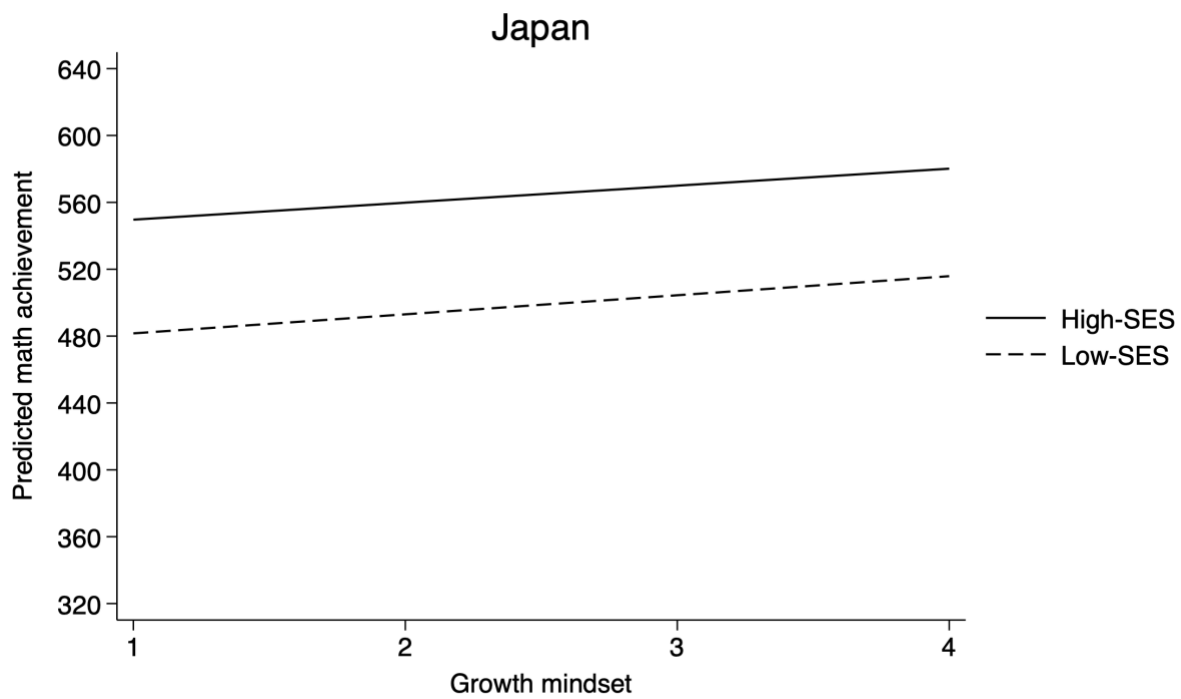

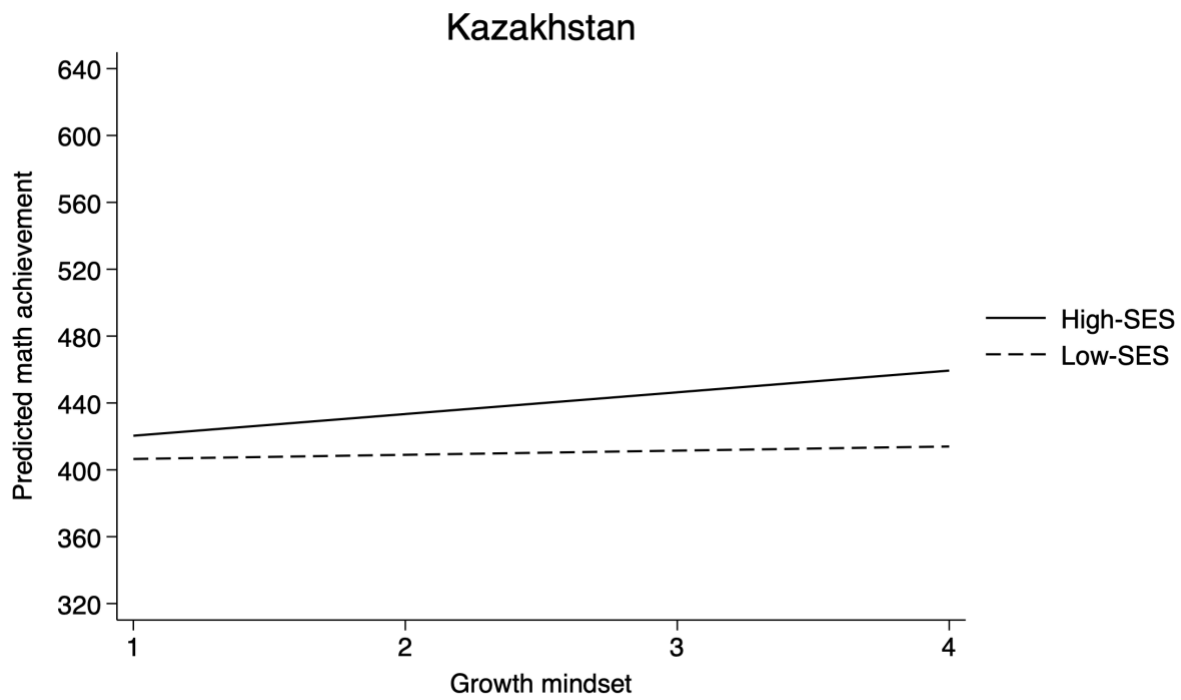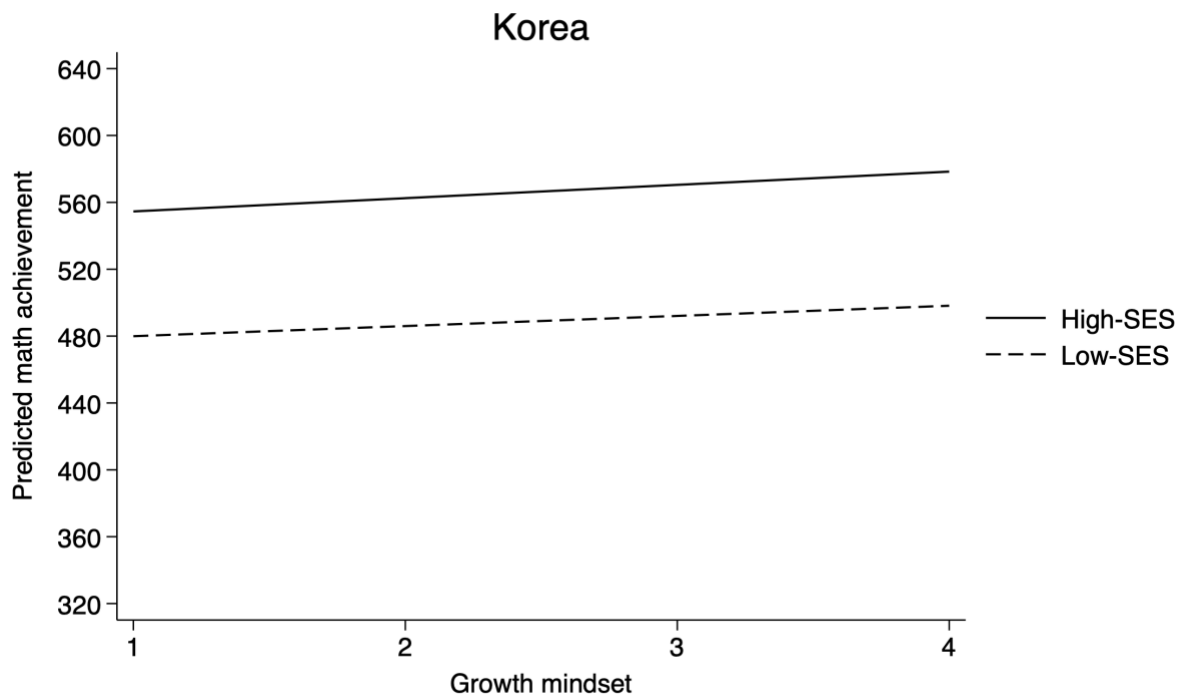

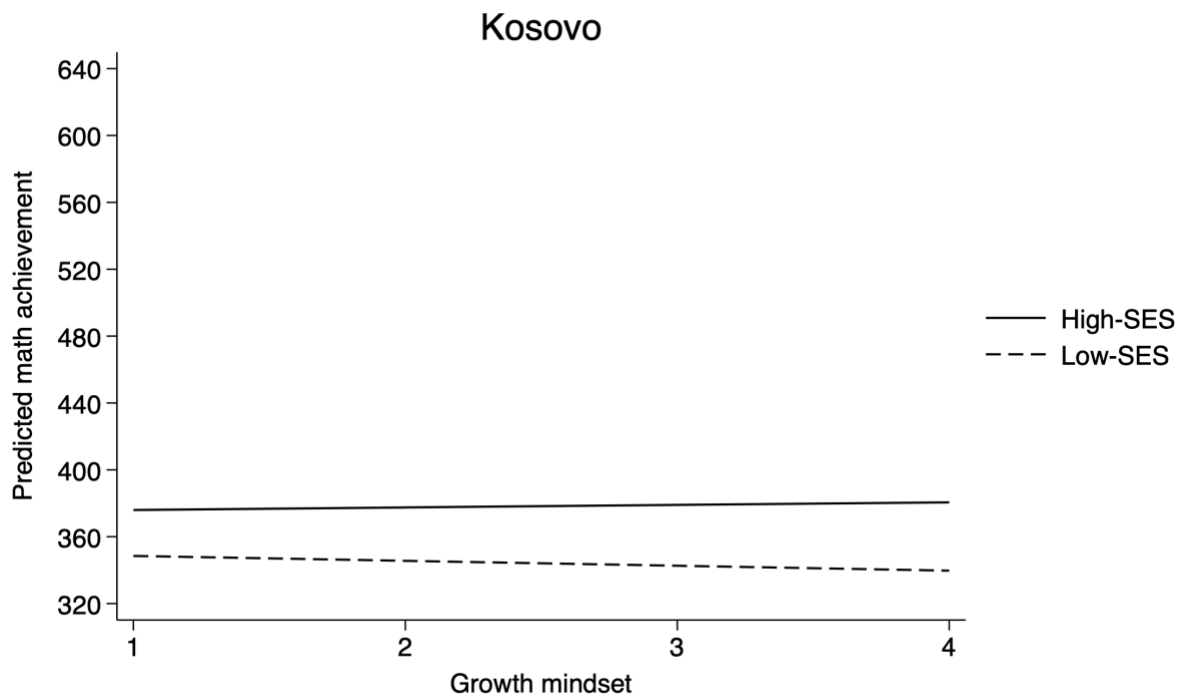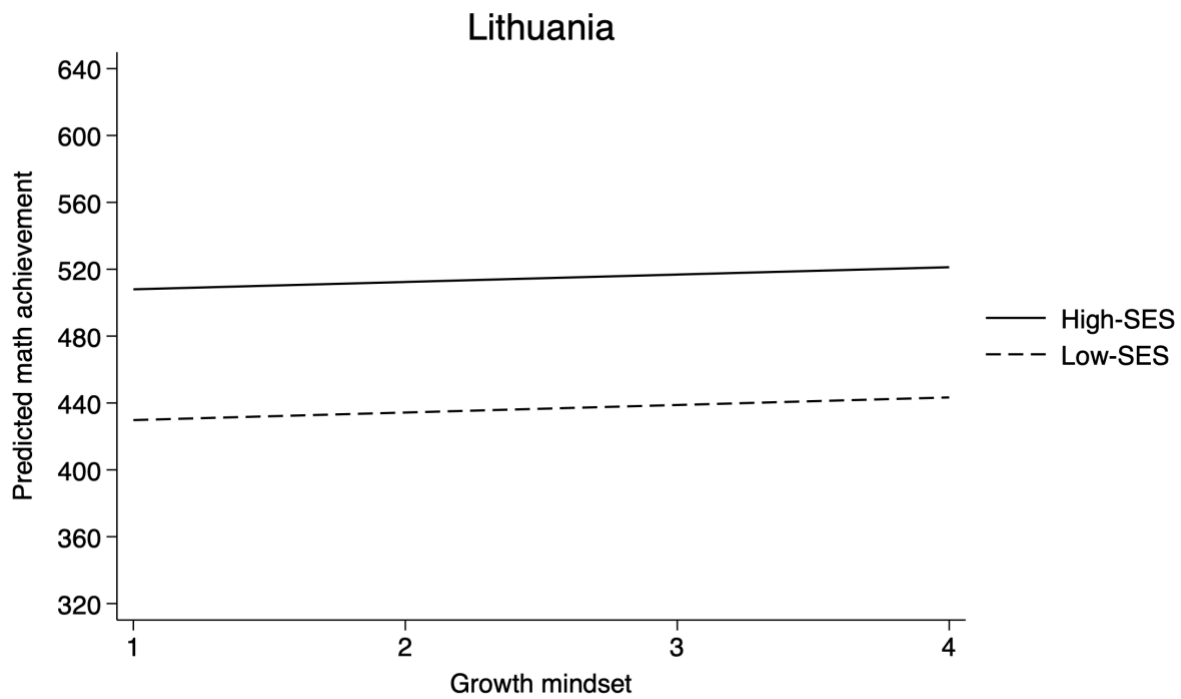

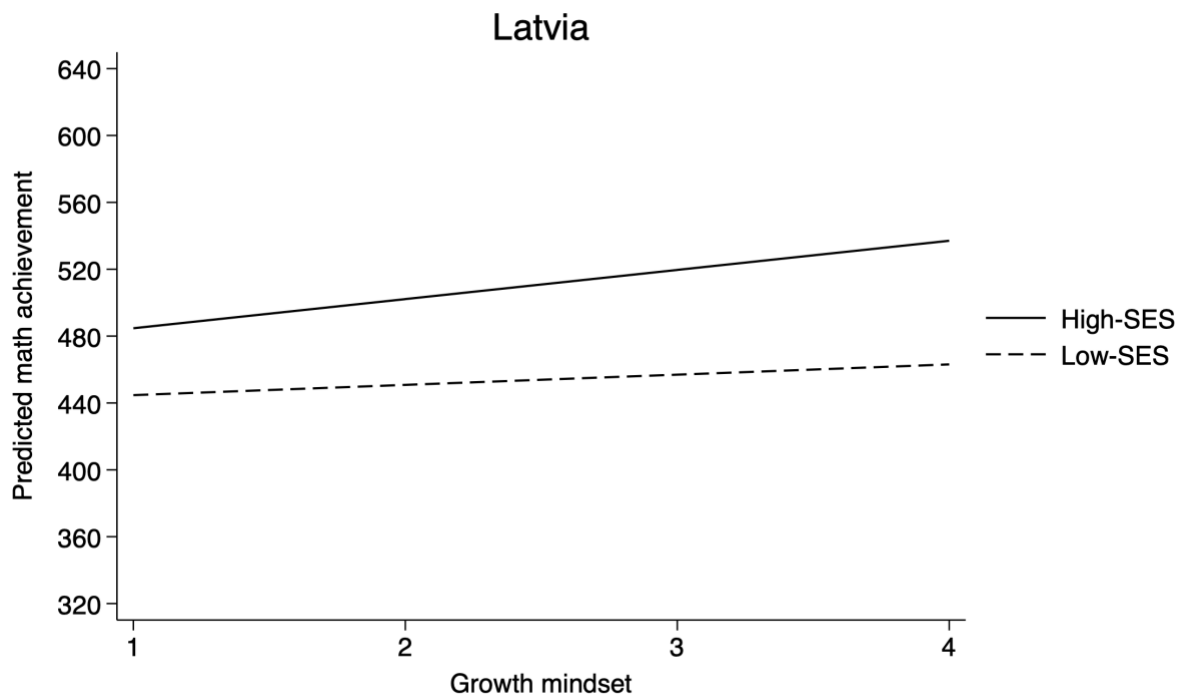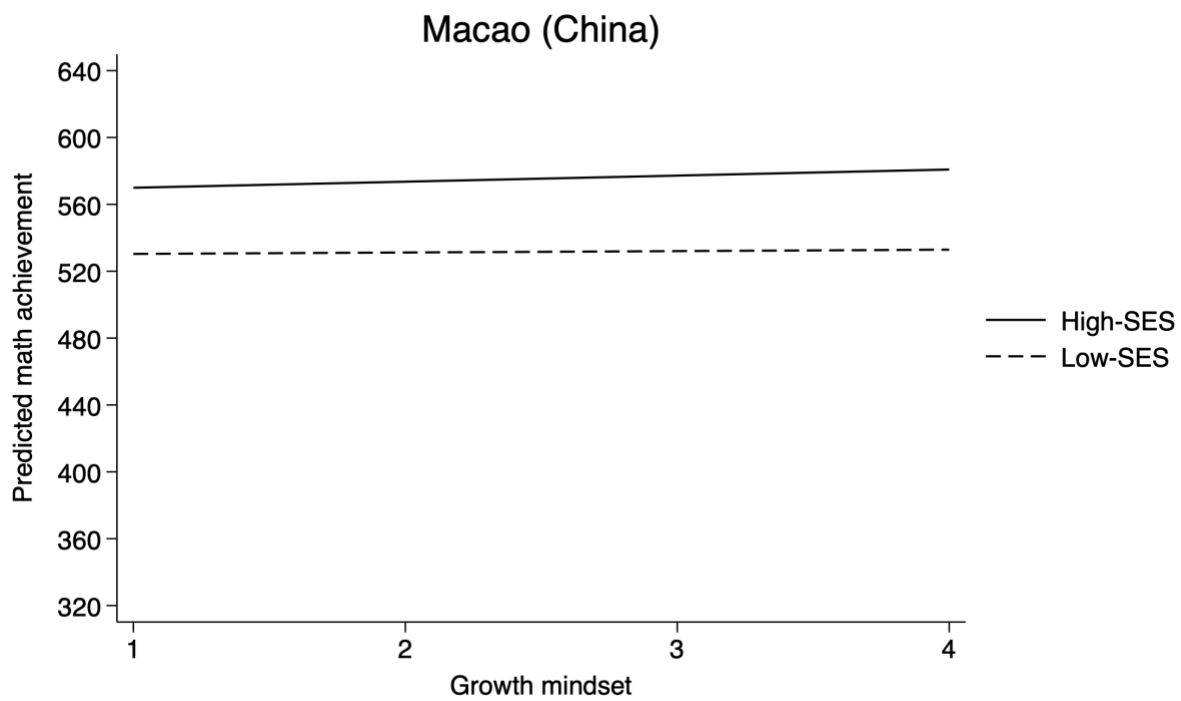

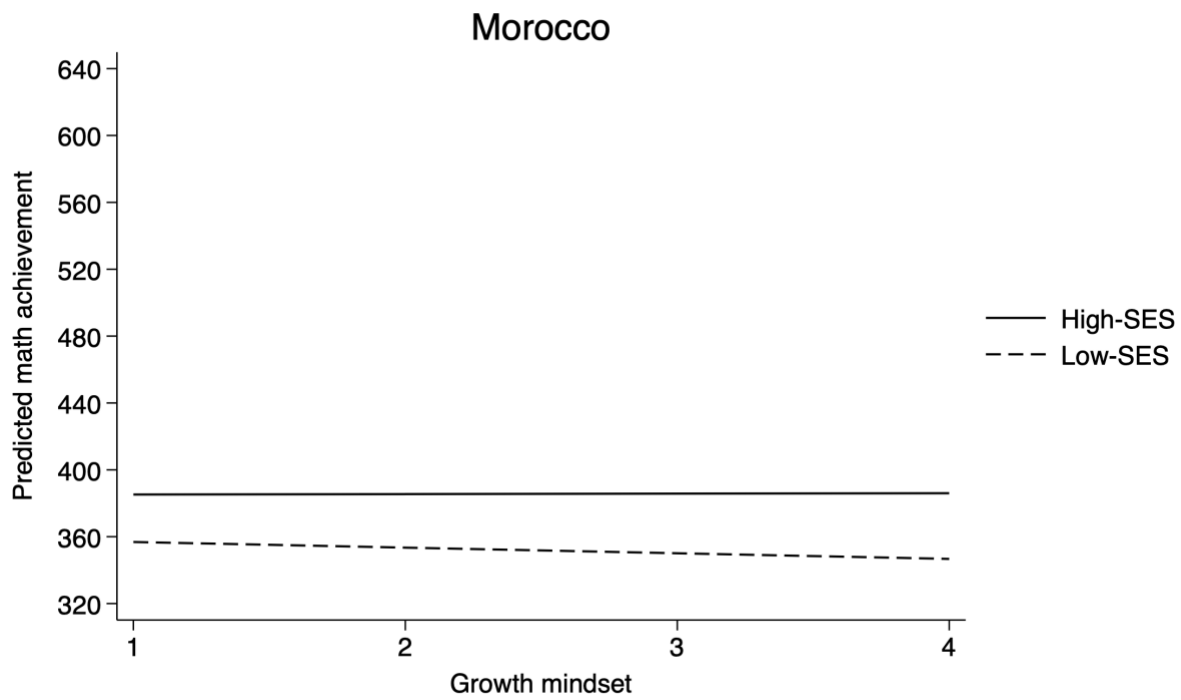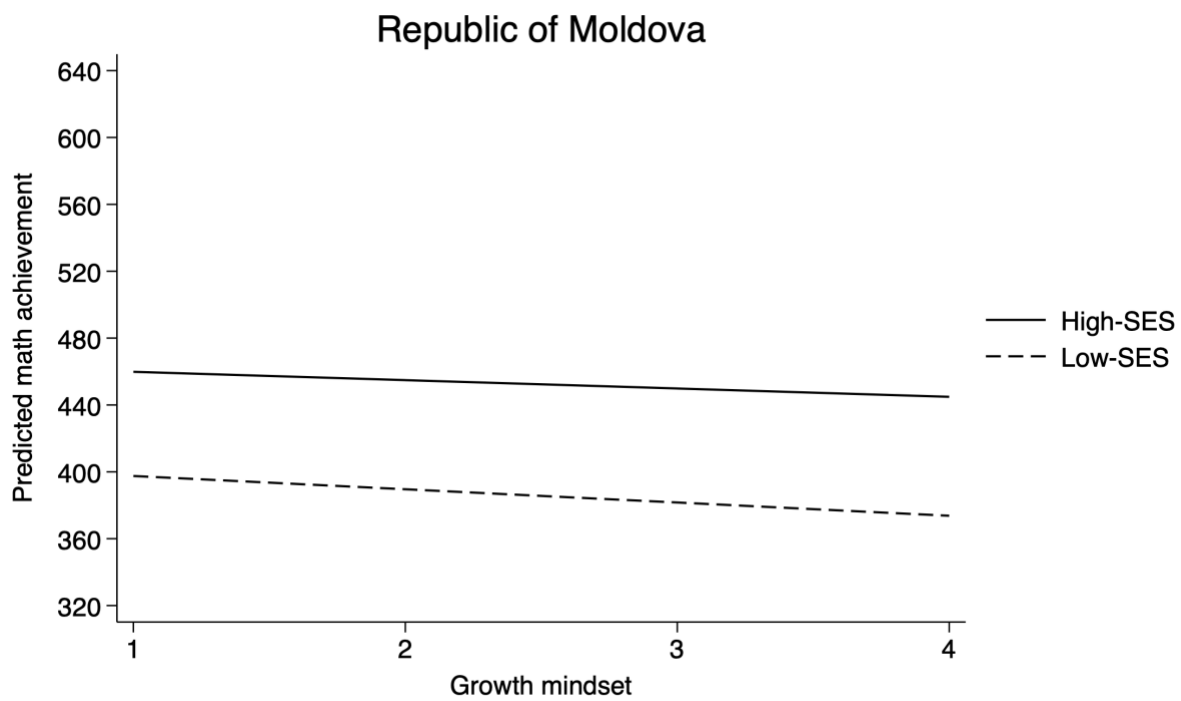

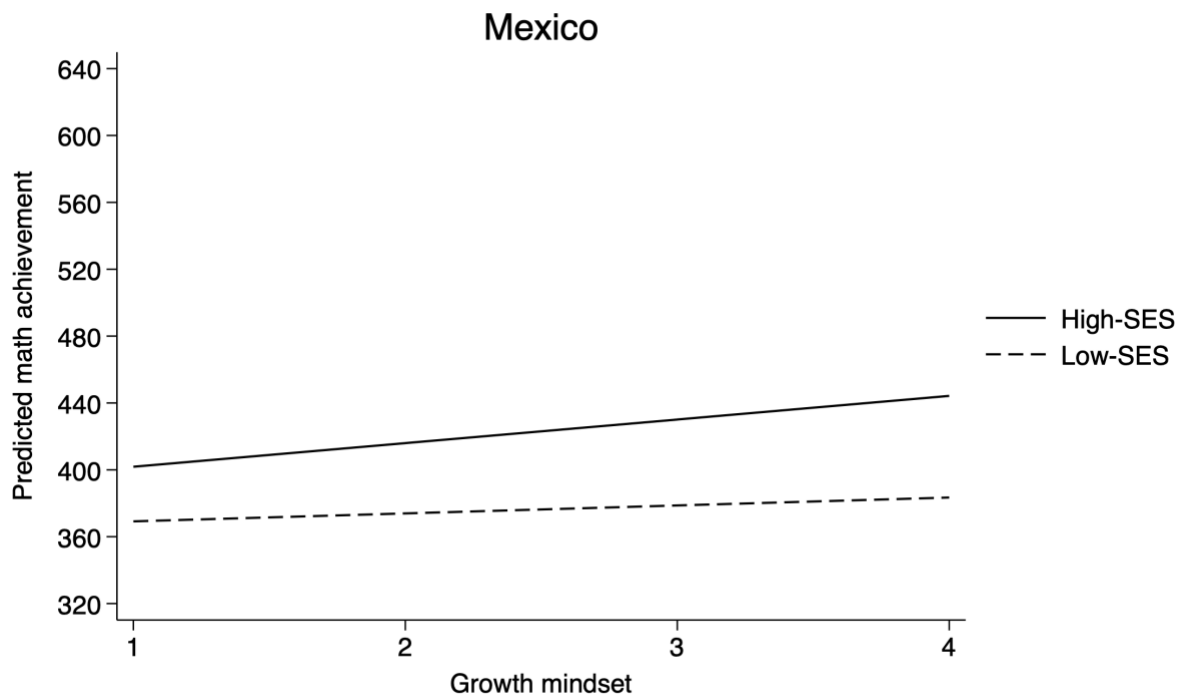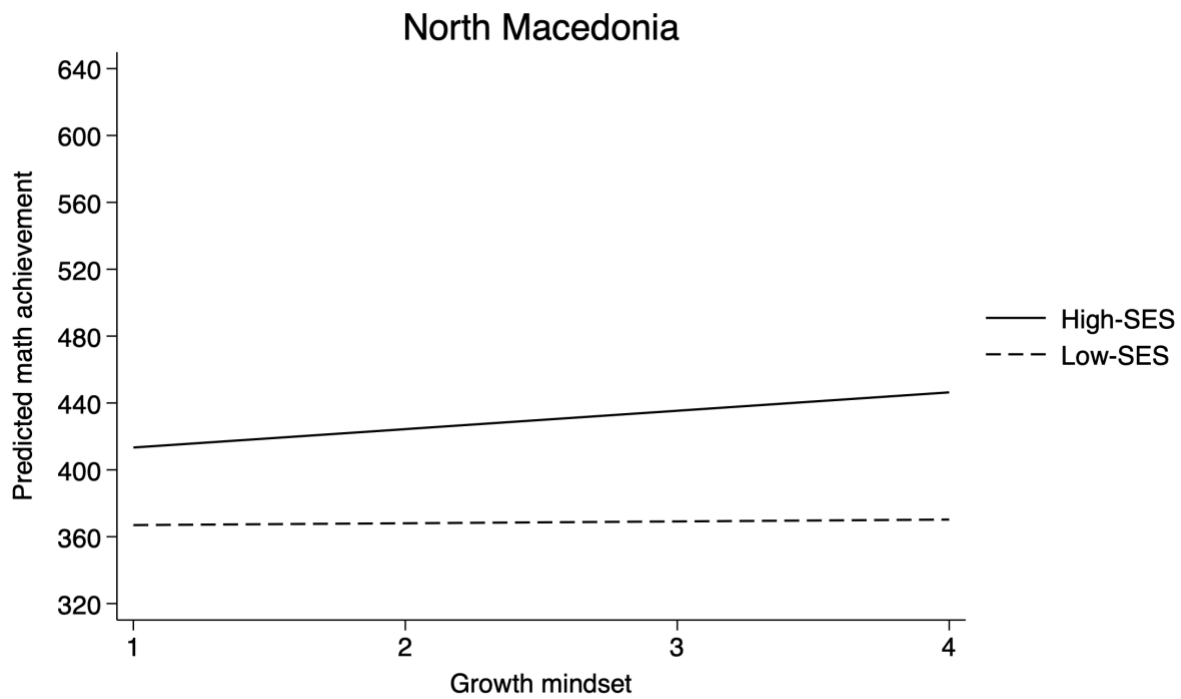

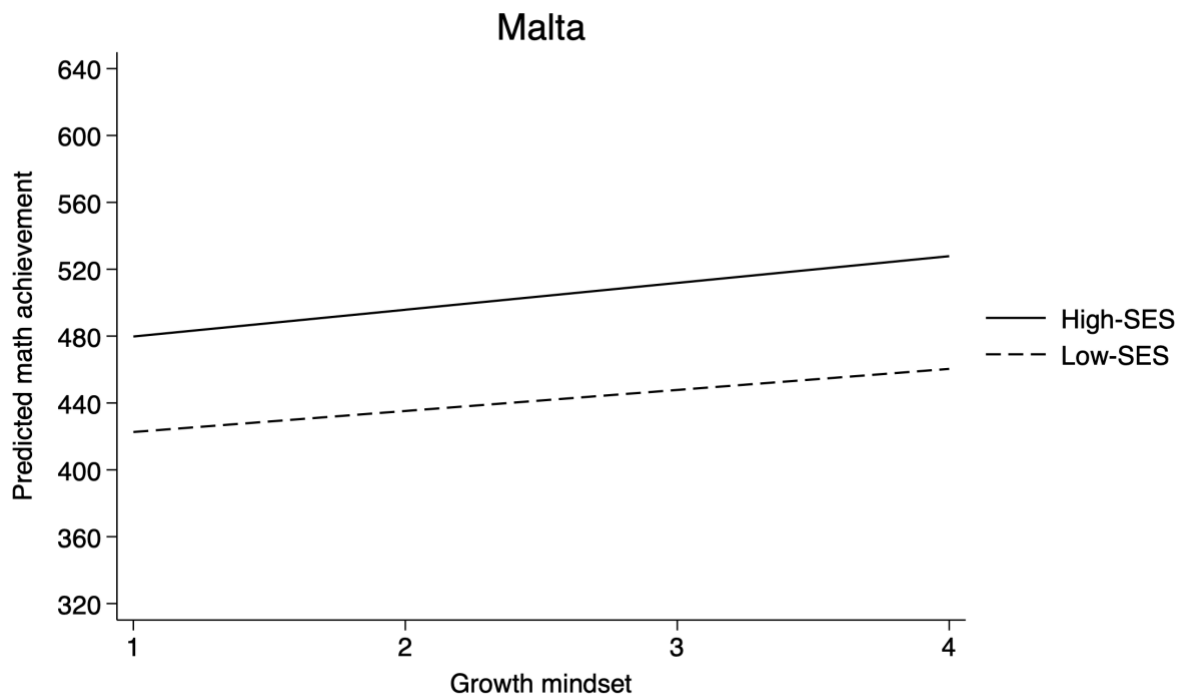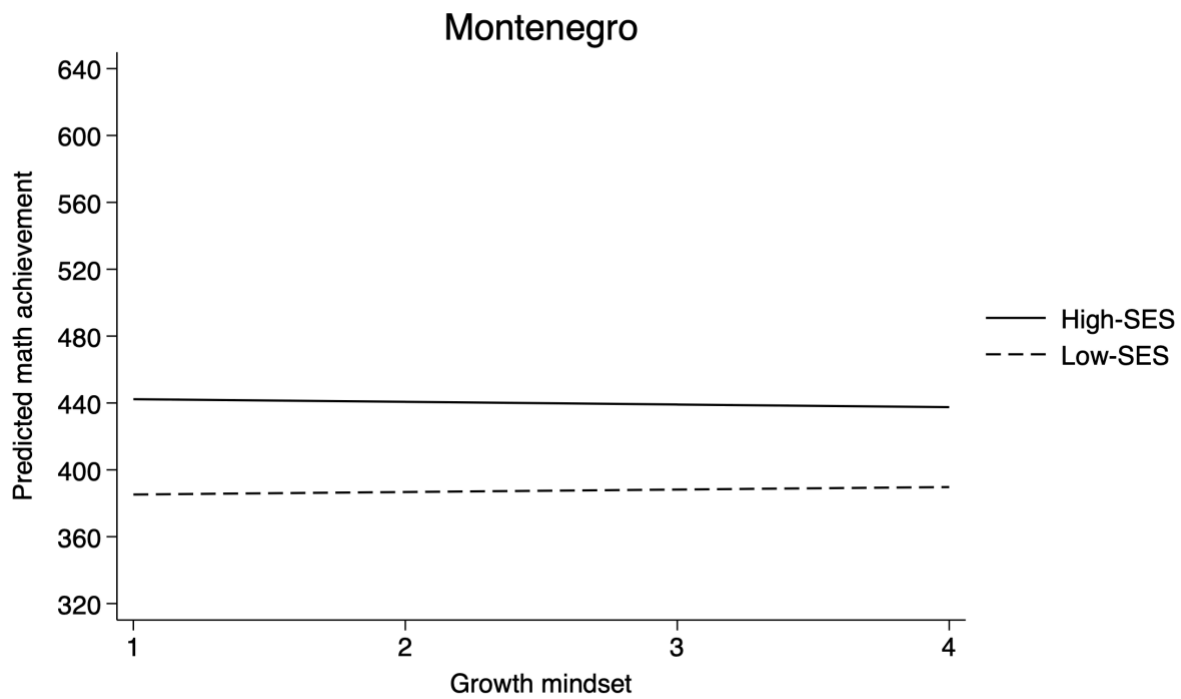

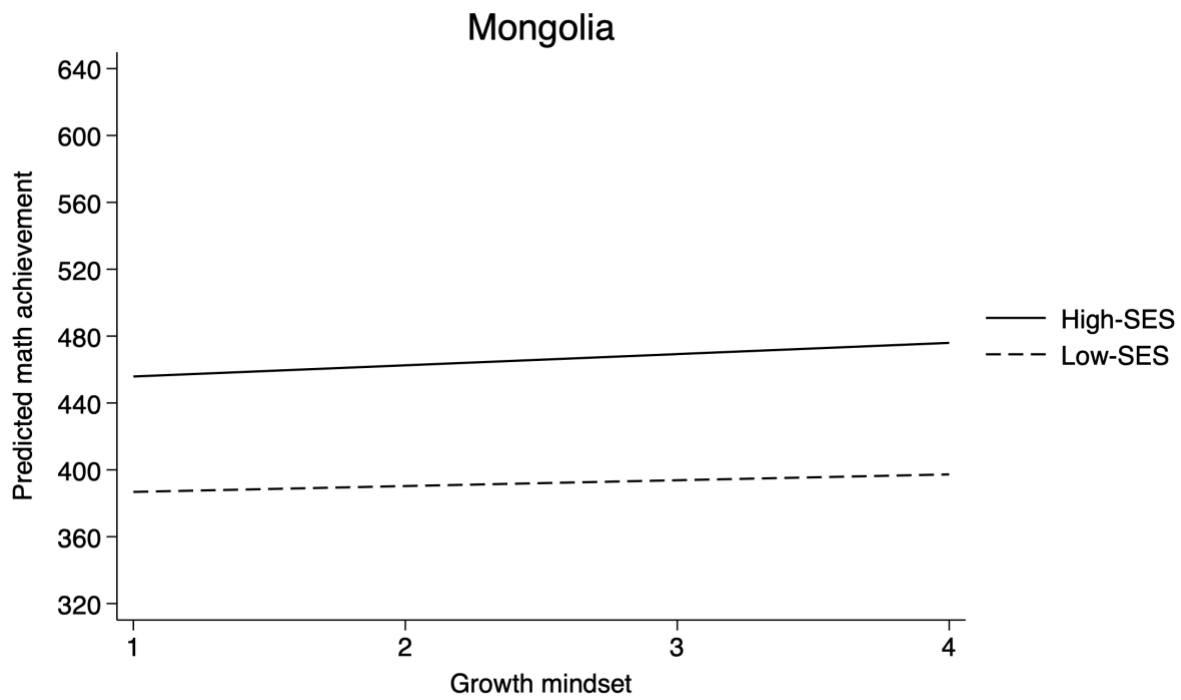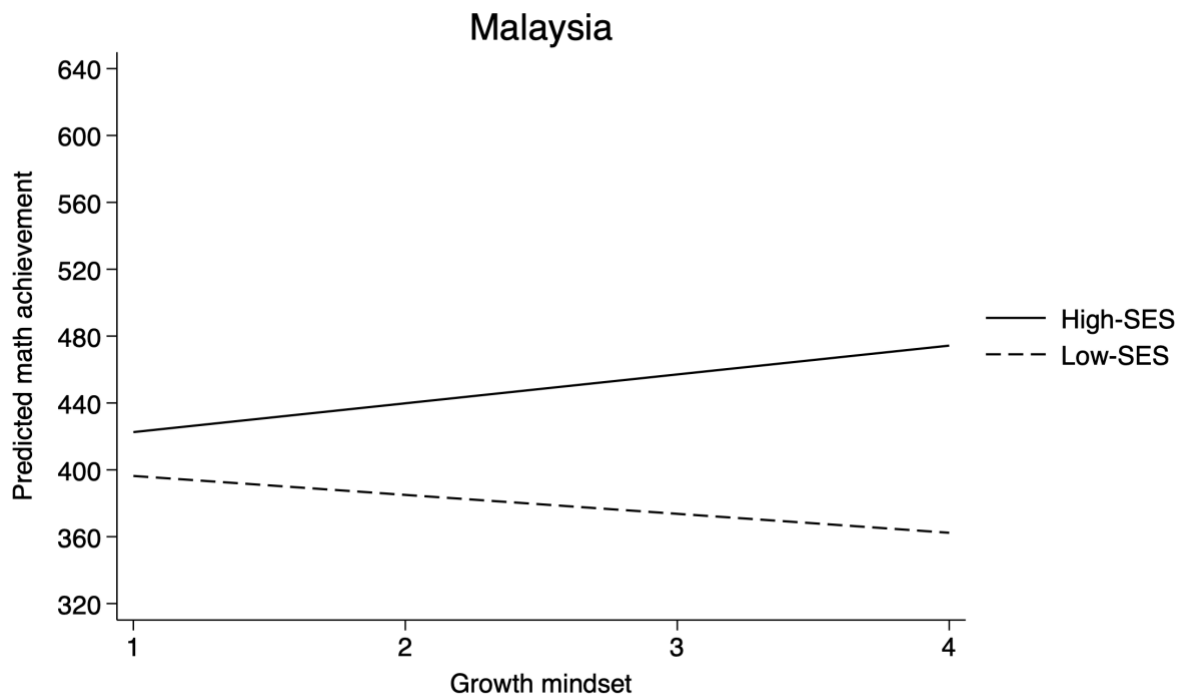

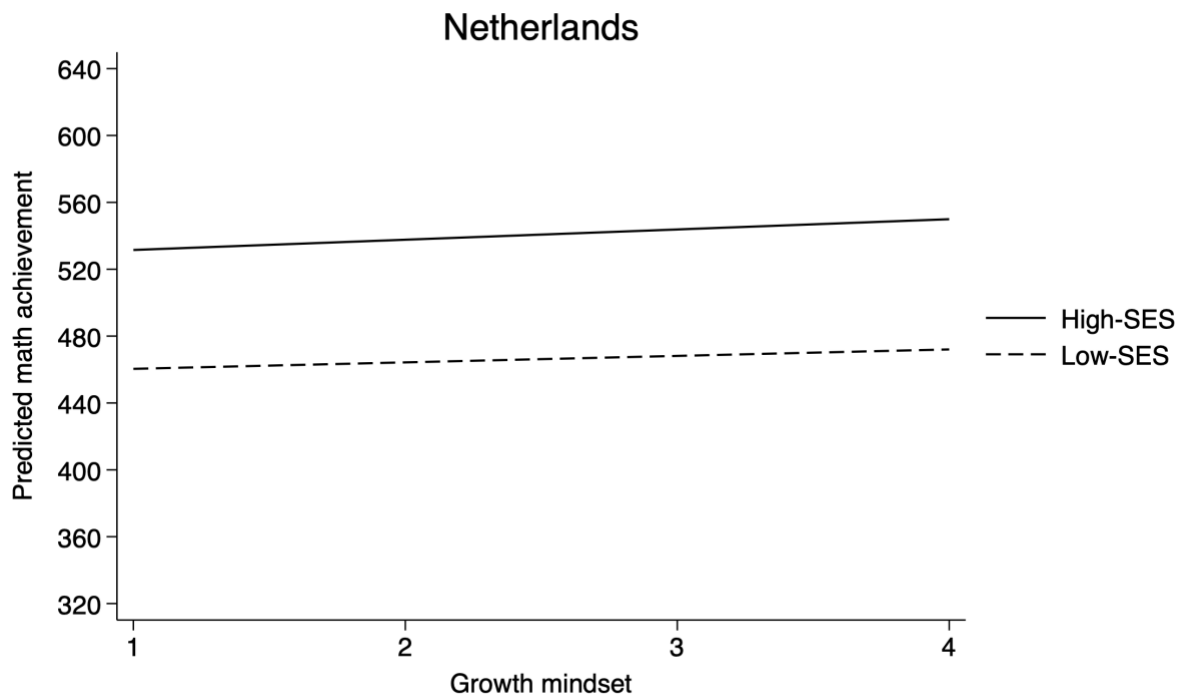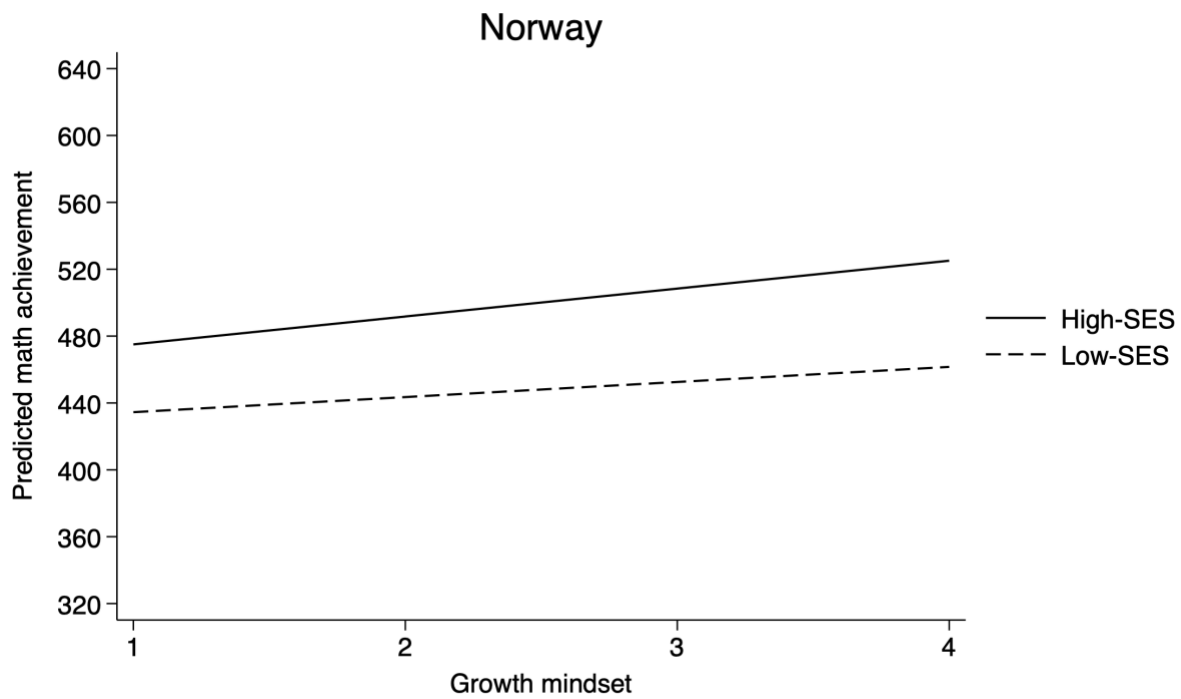

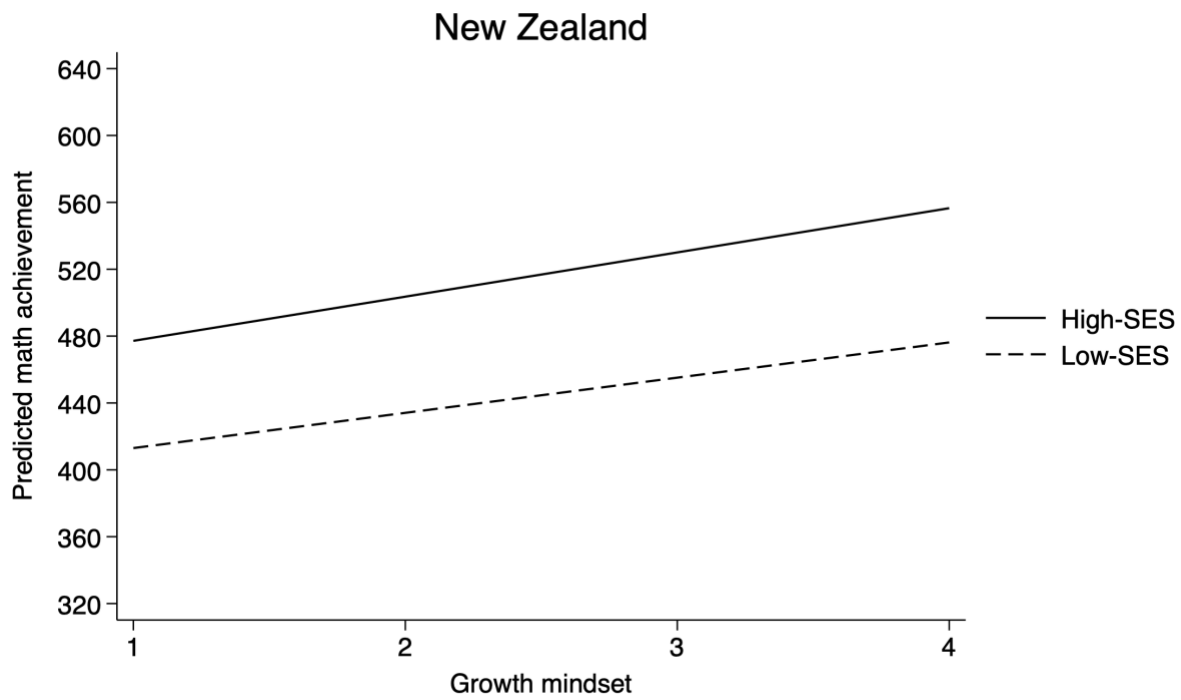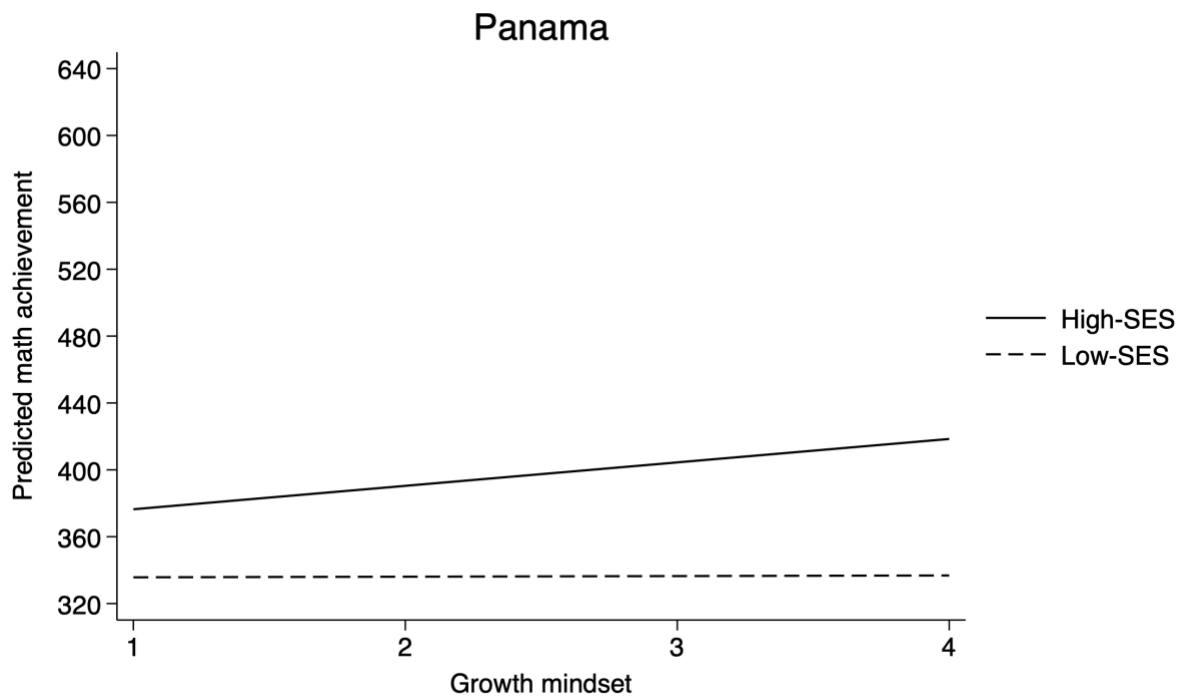

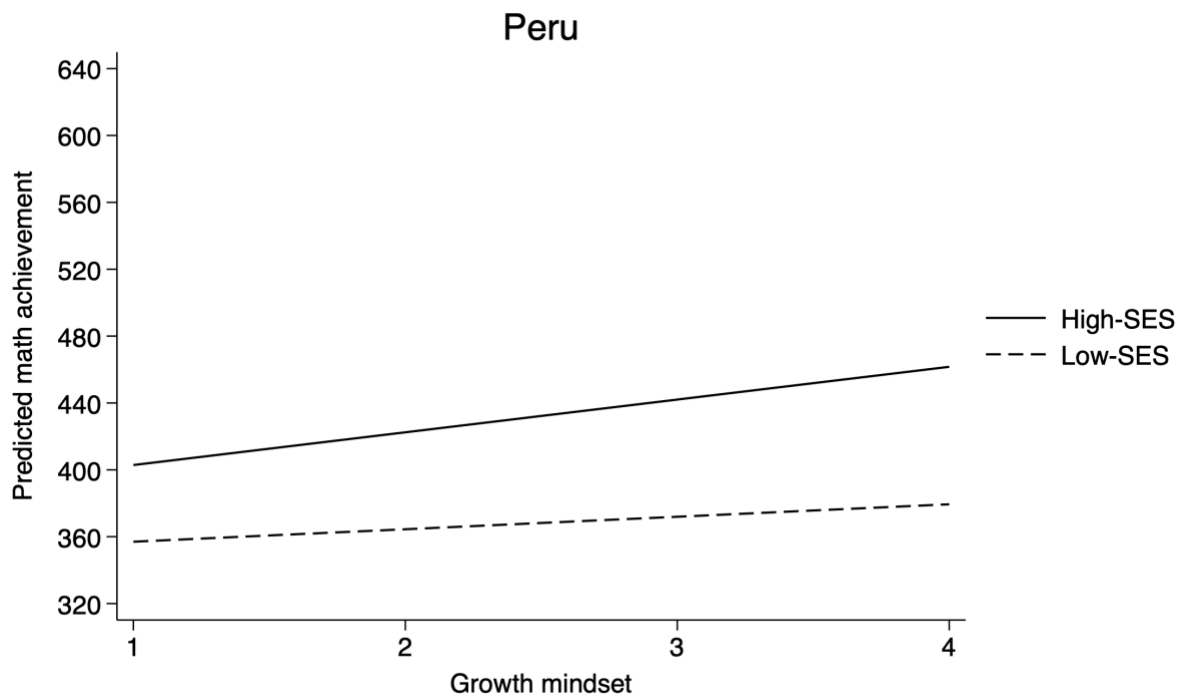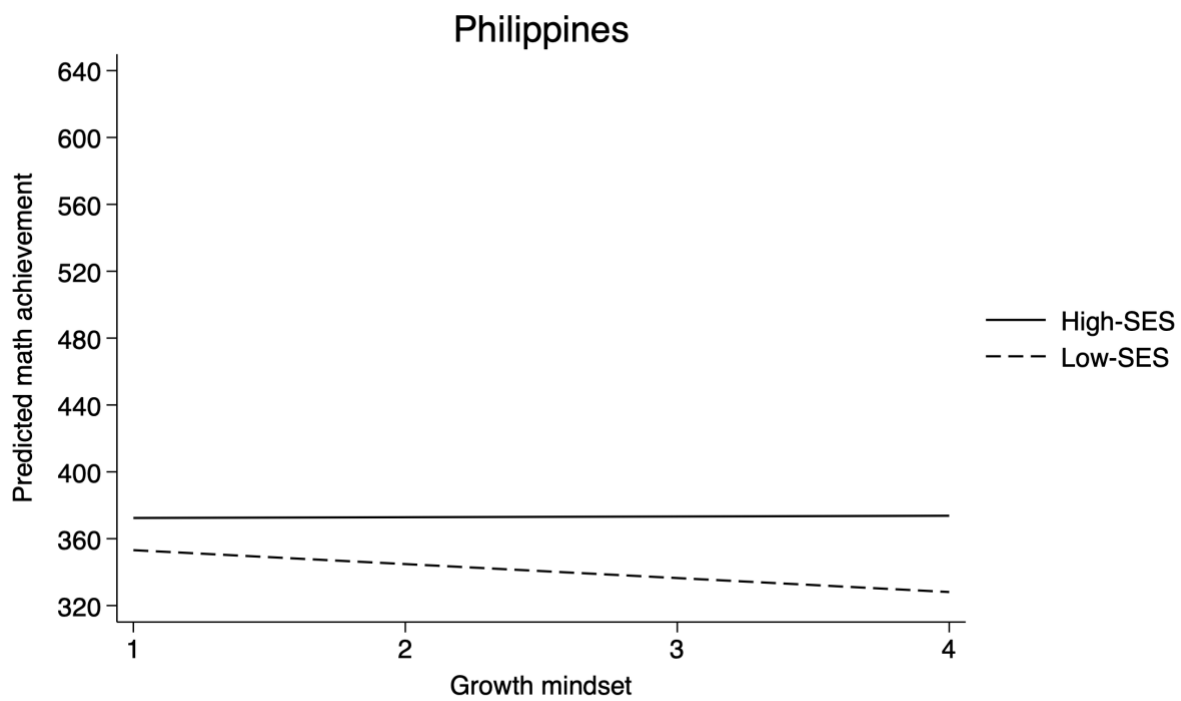

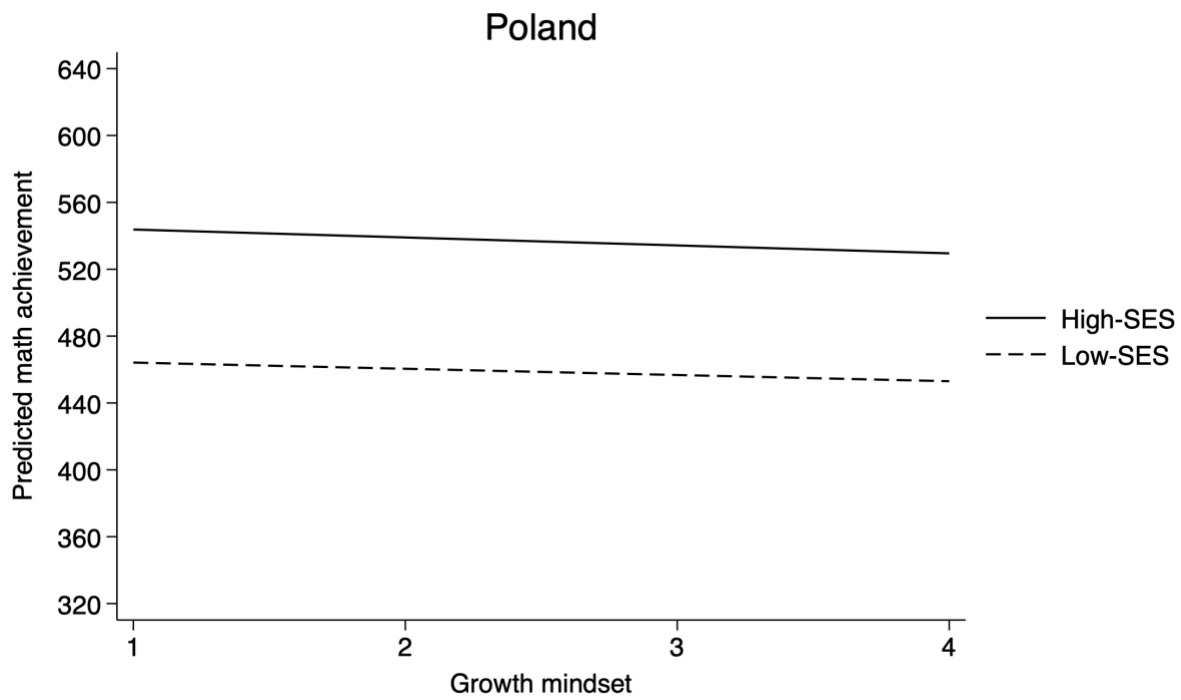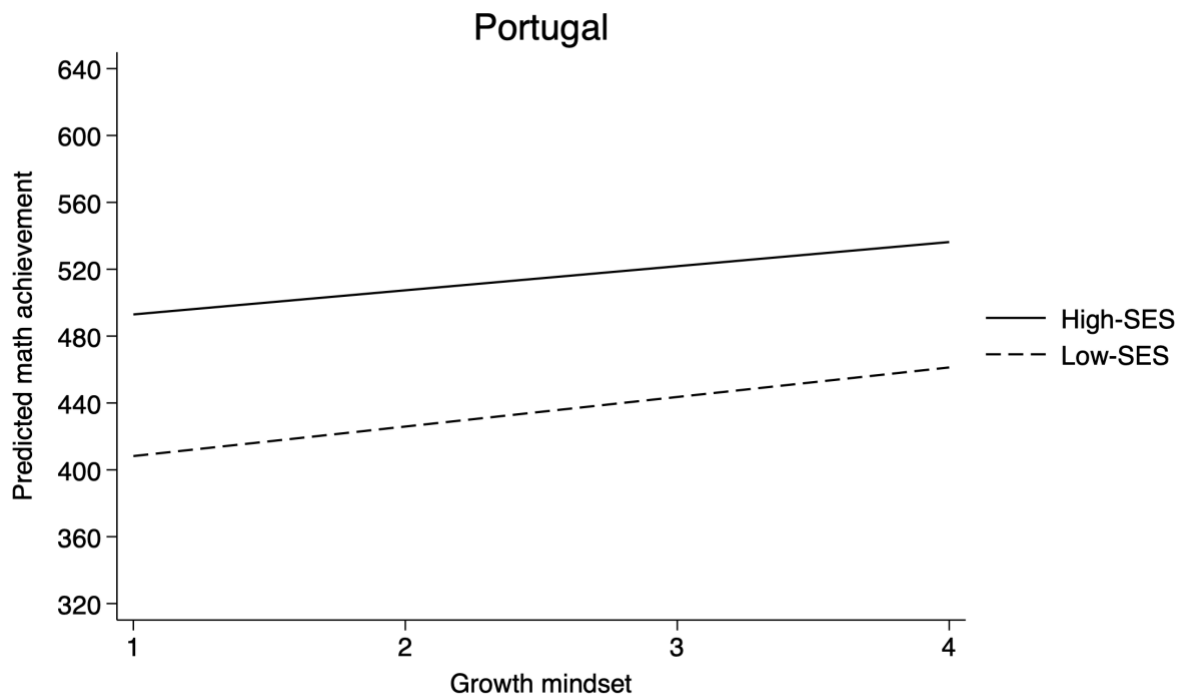

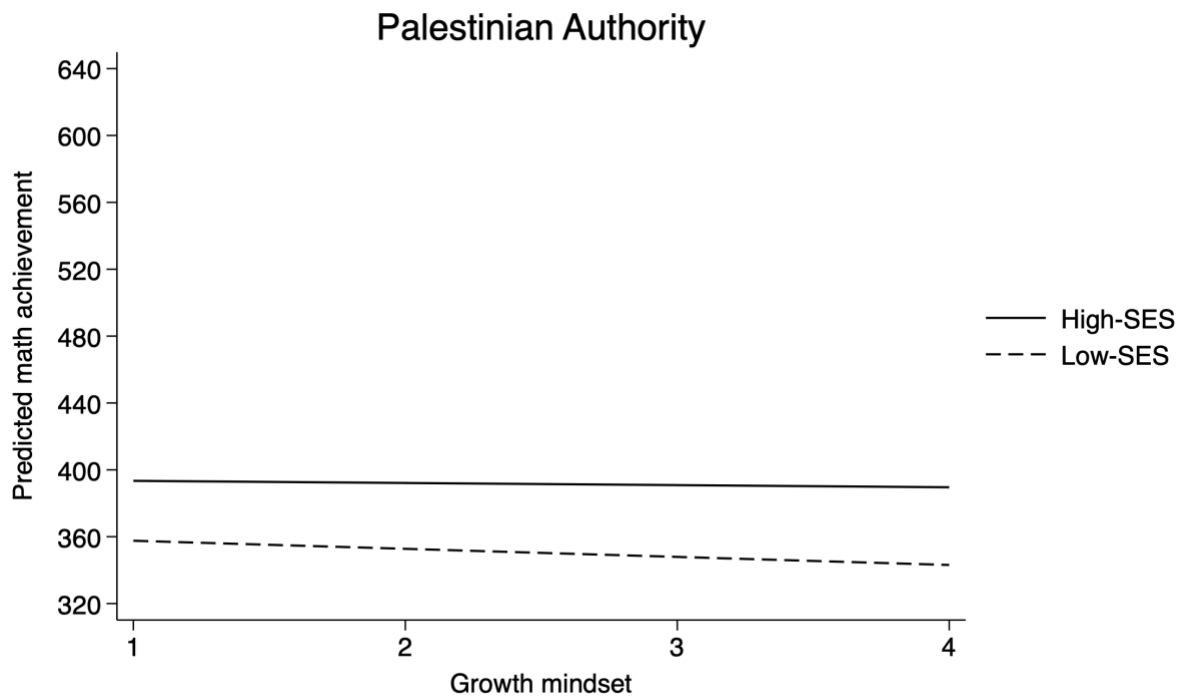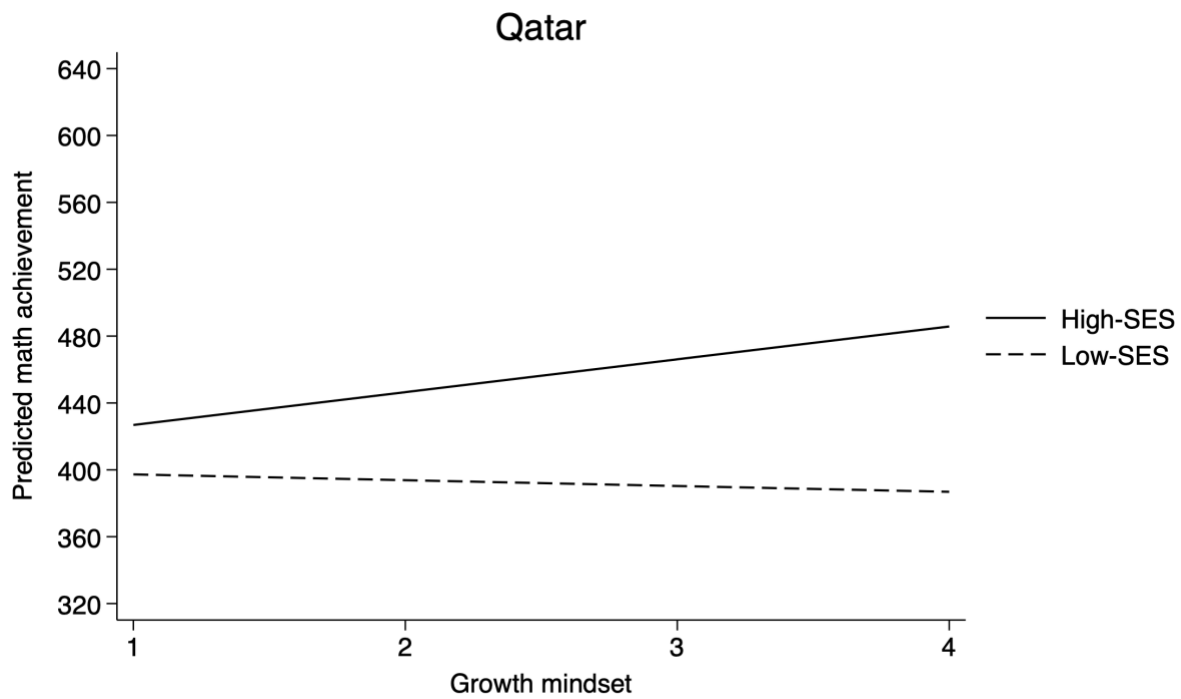

### Baku (Azerbaijan)

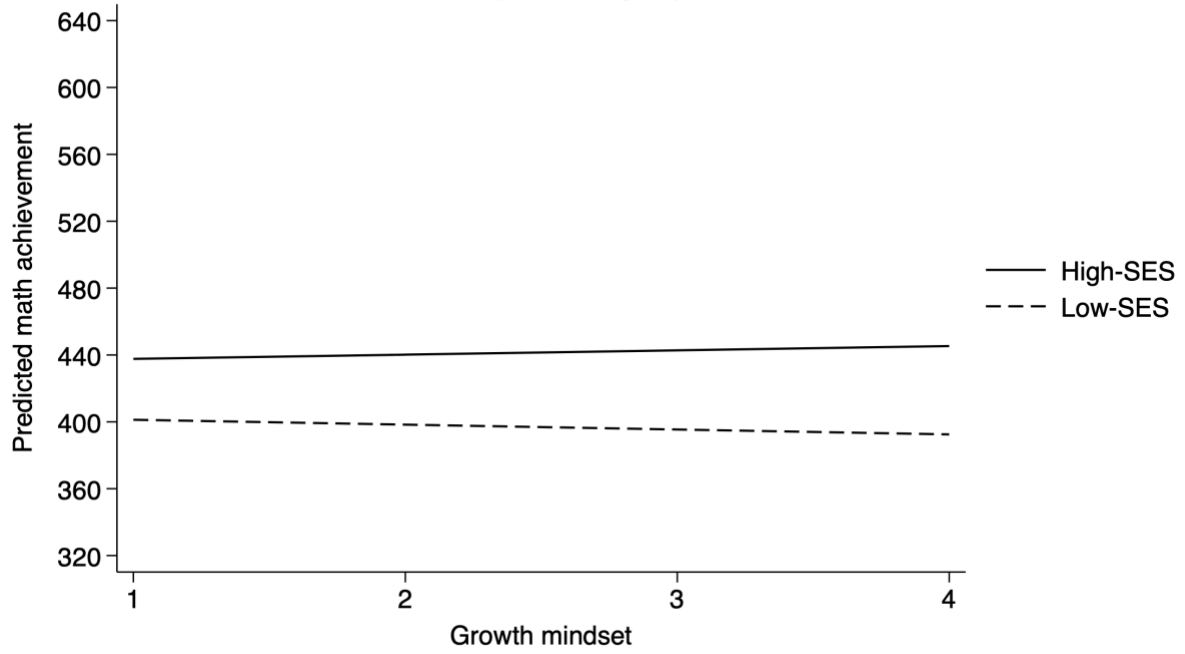

### Ukrainian regions (18 of 27)

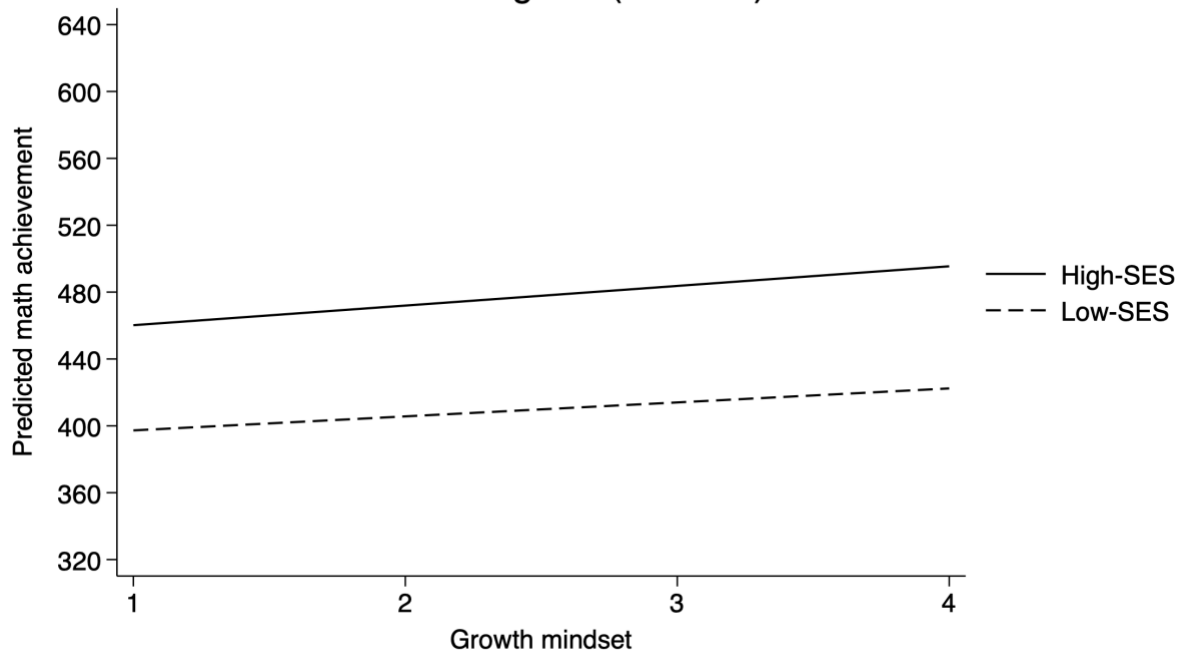

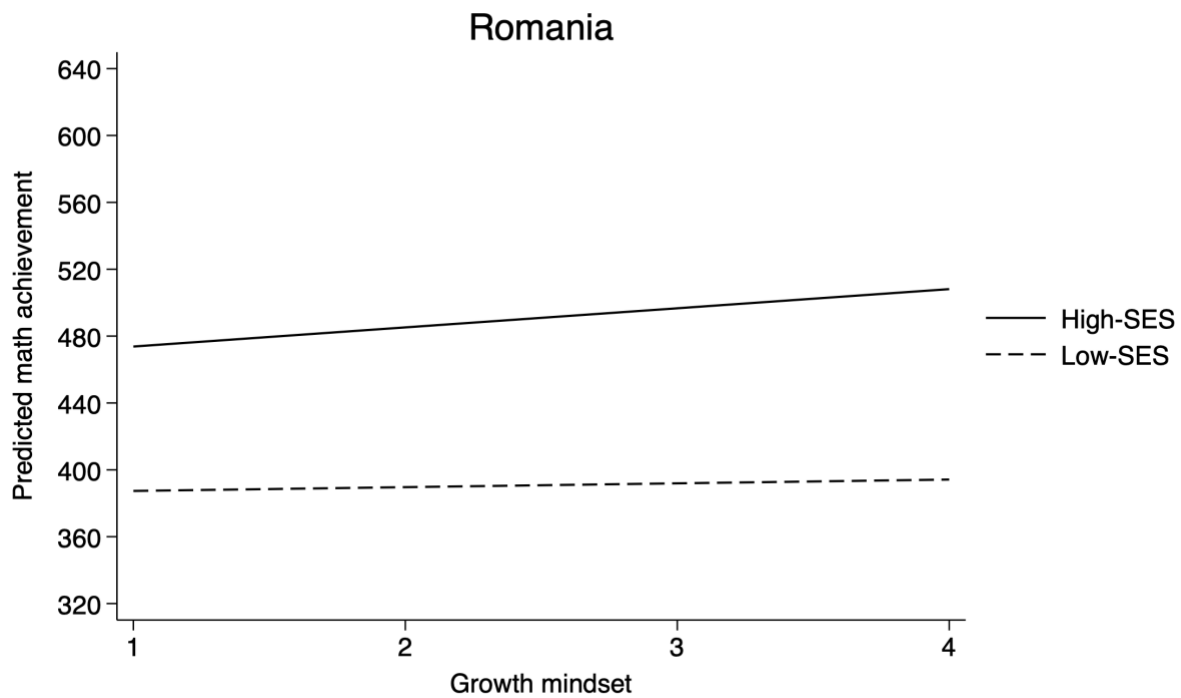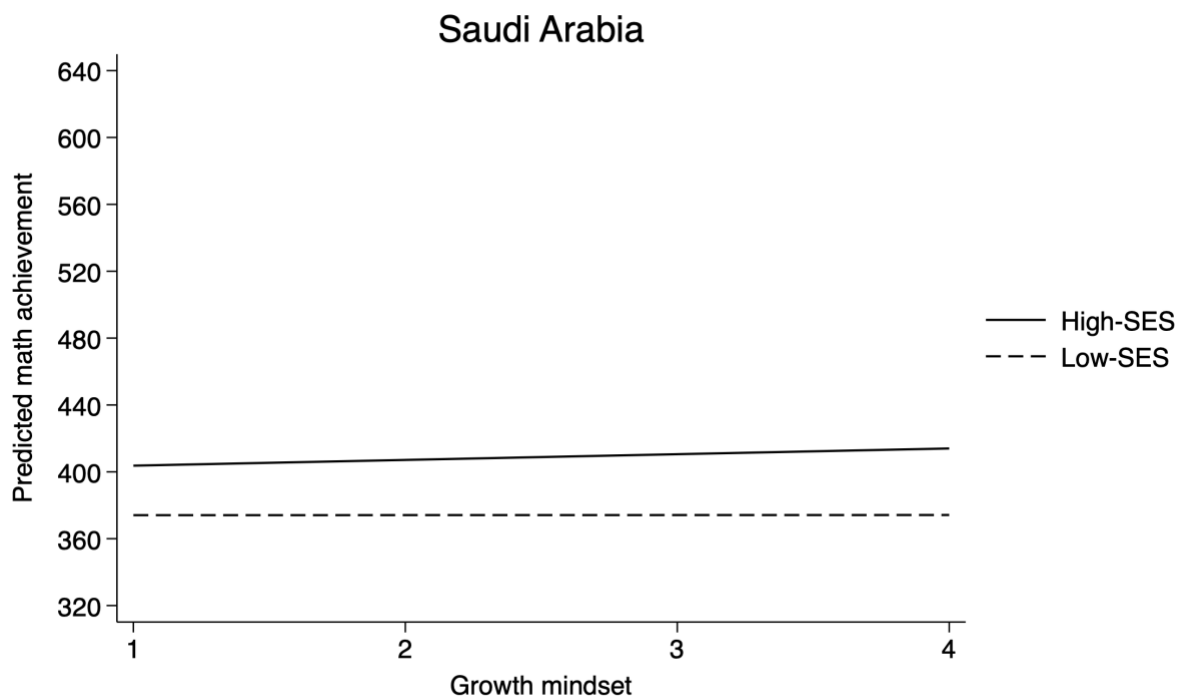

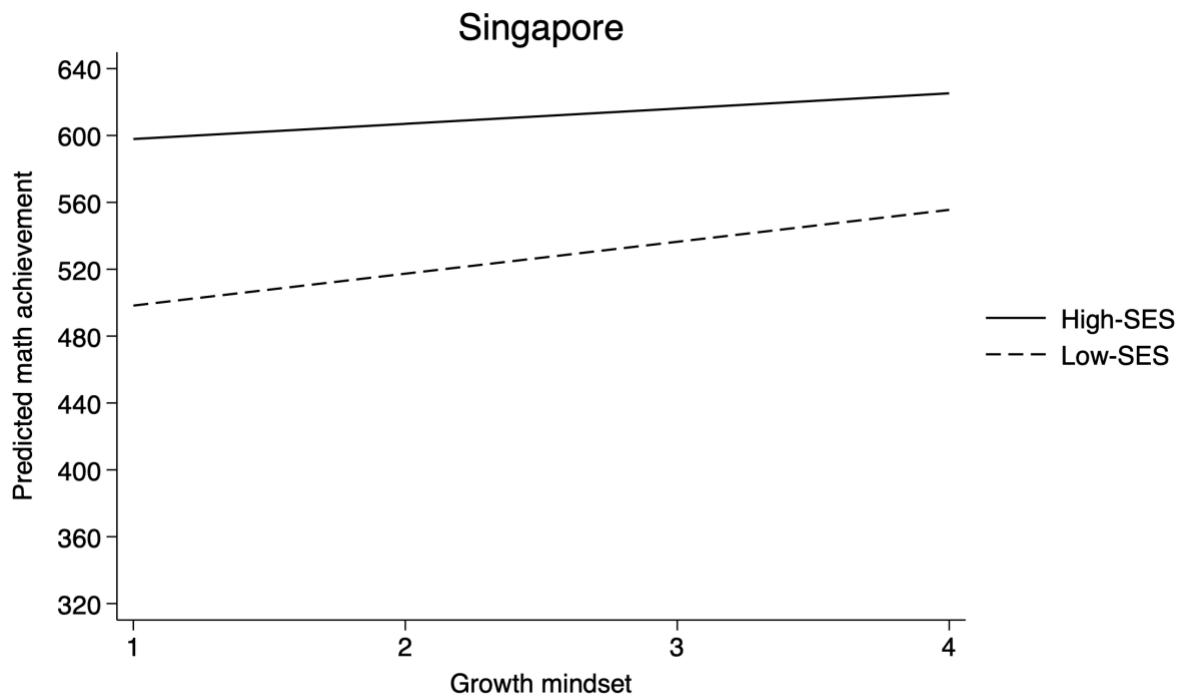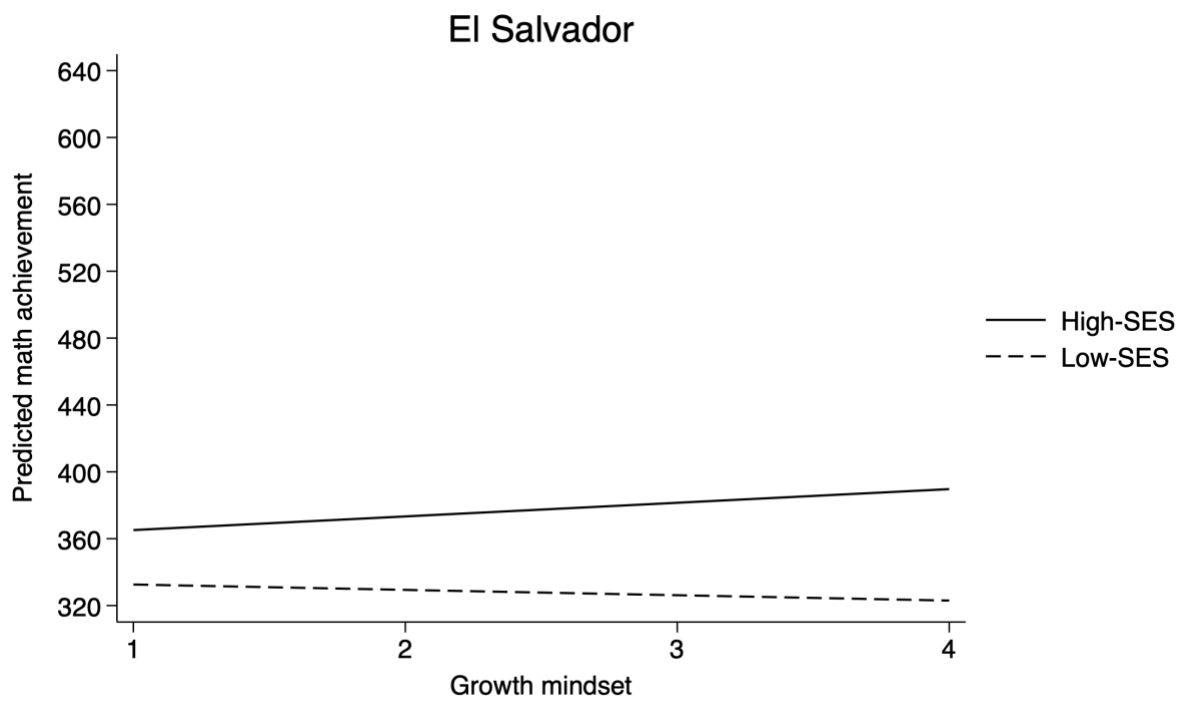

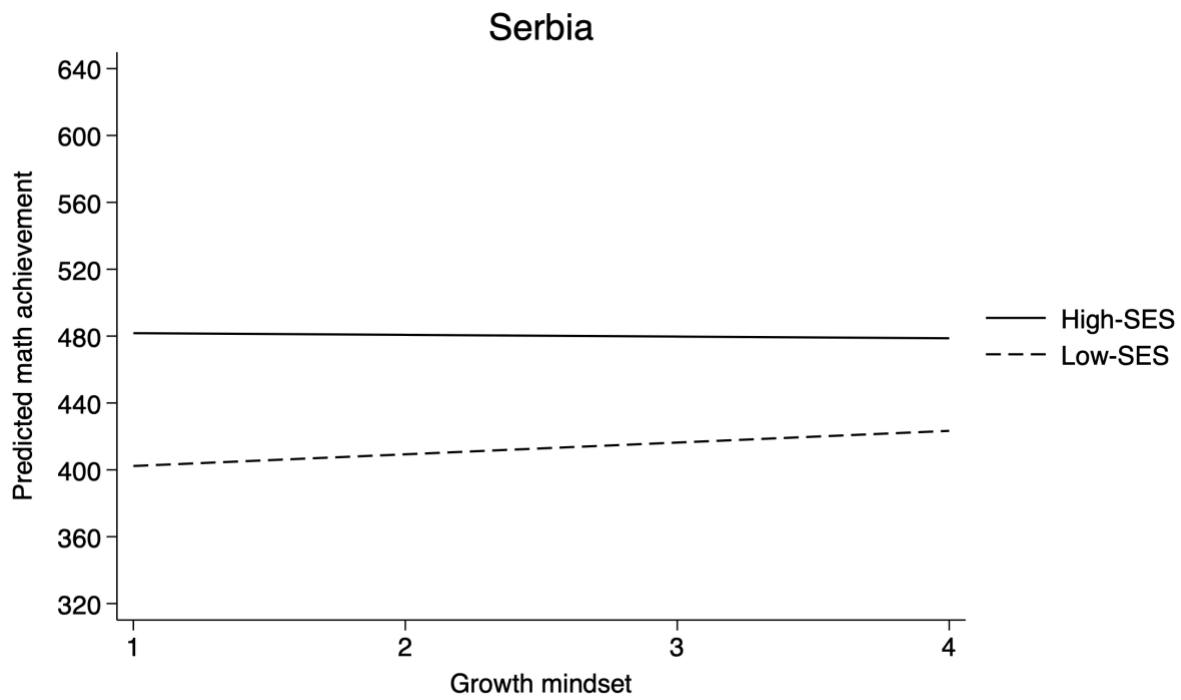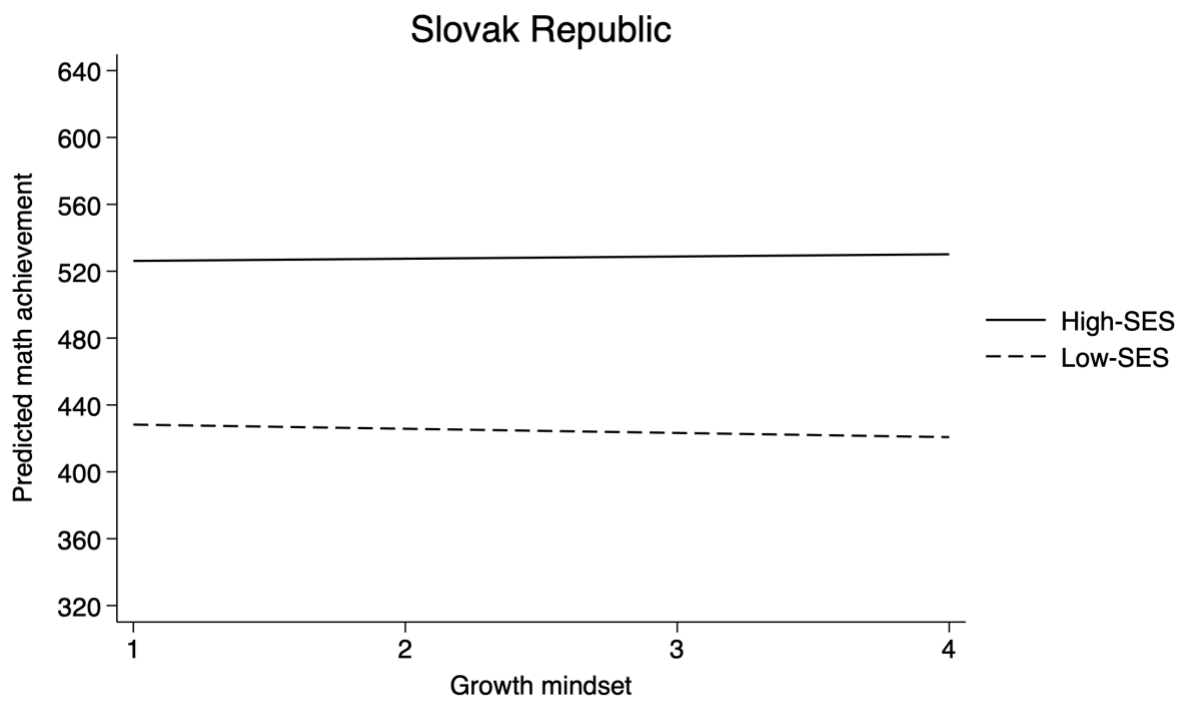

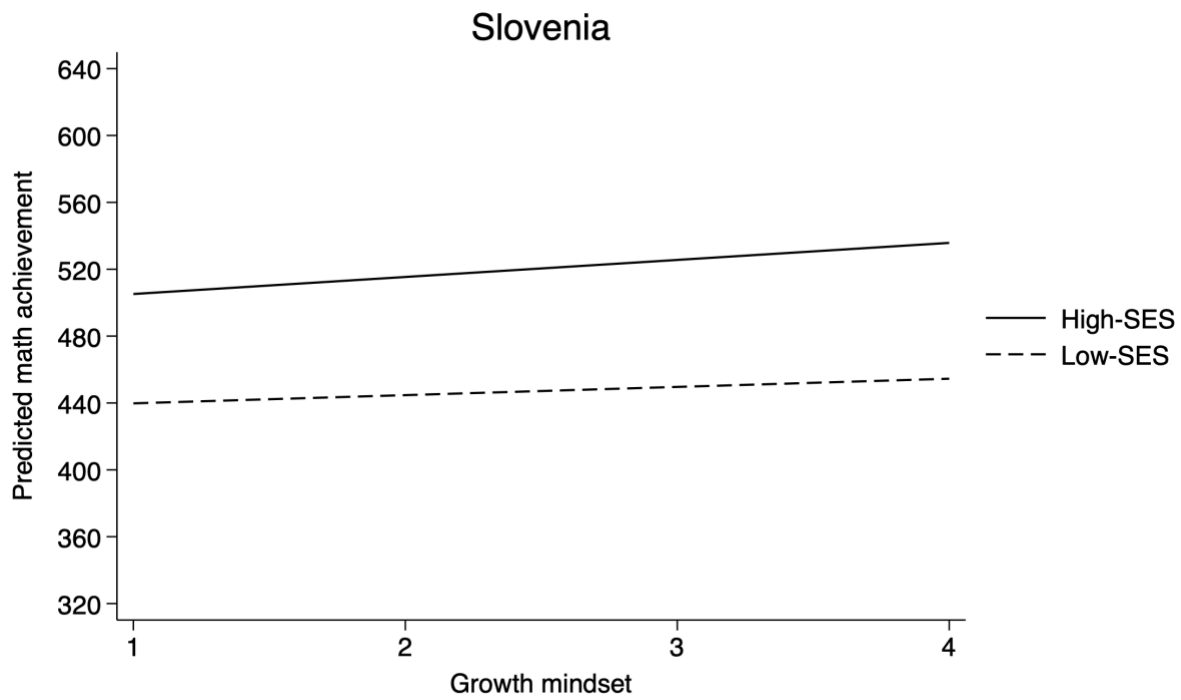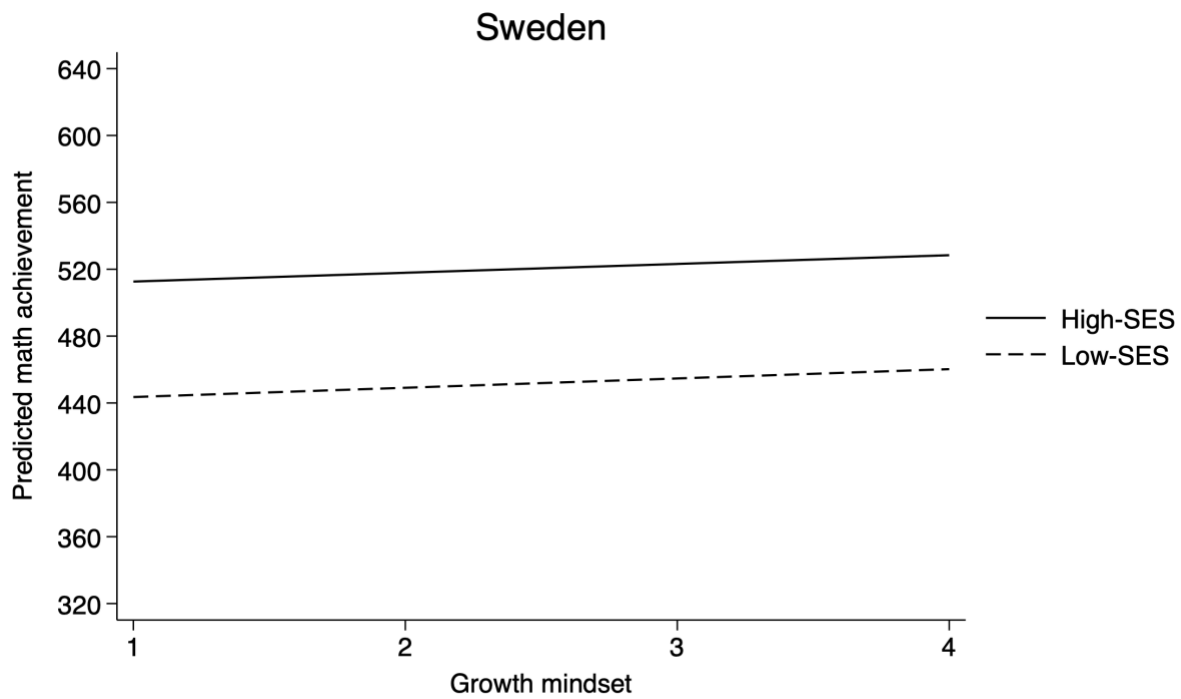

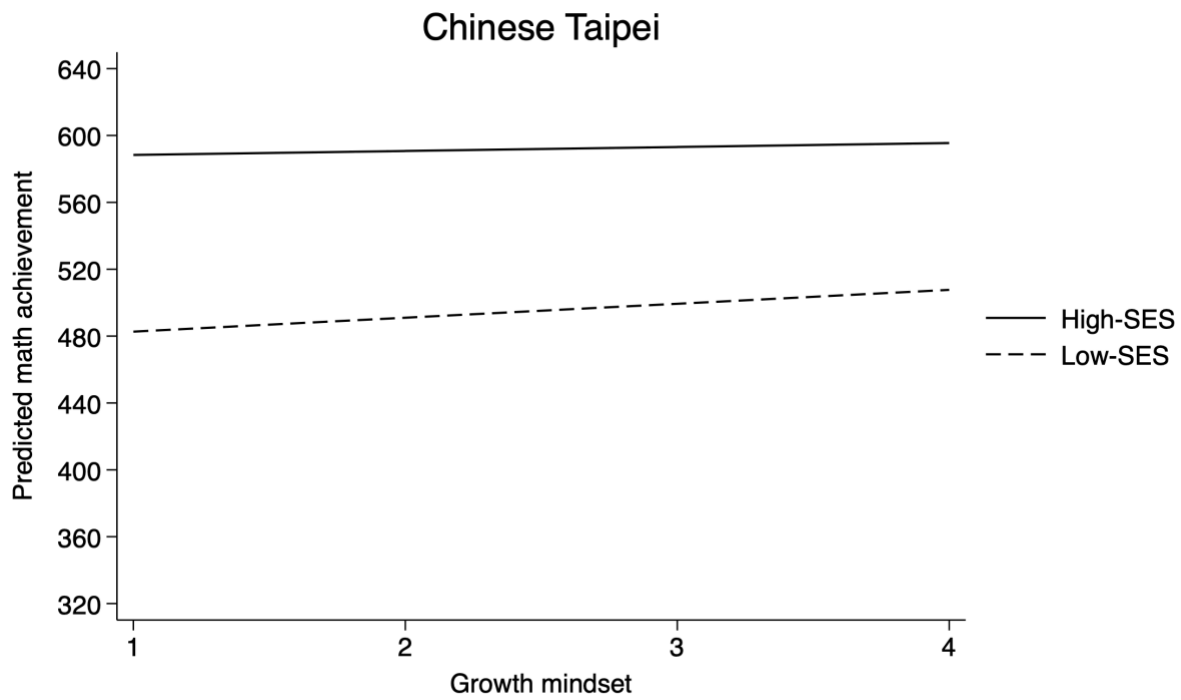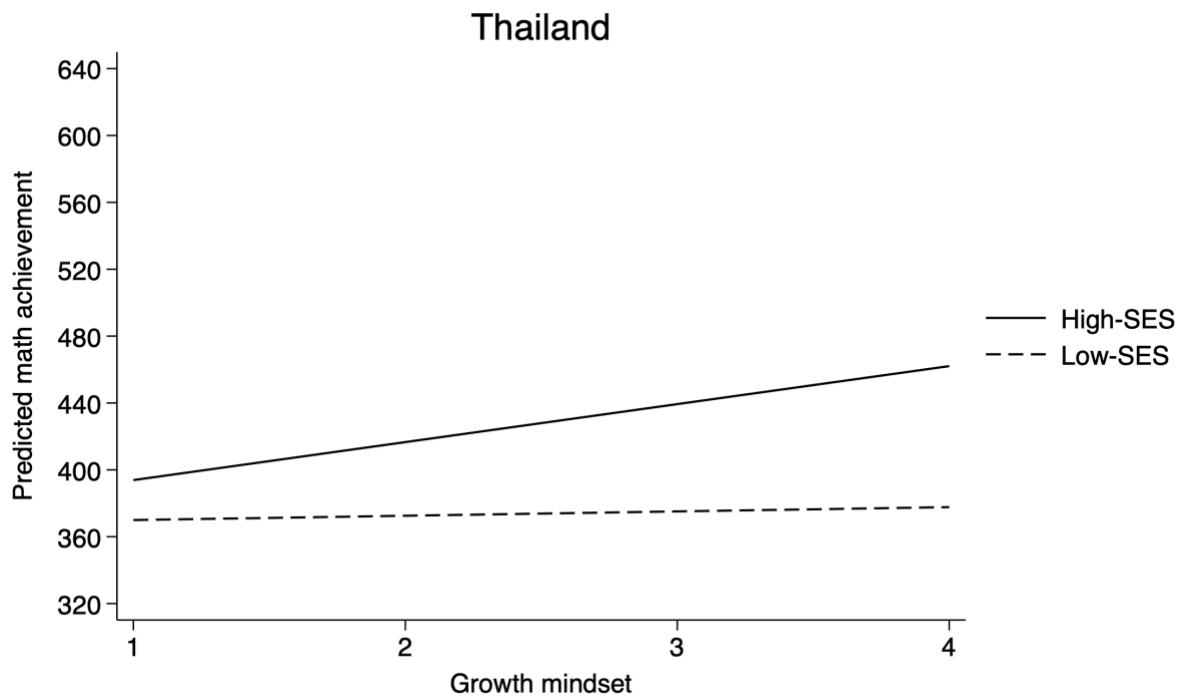

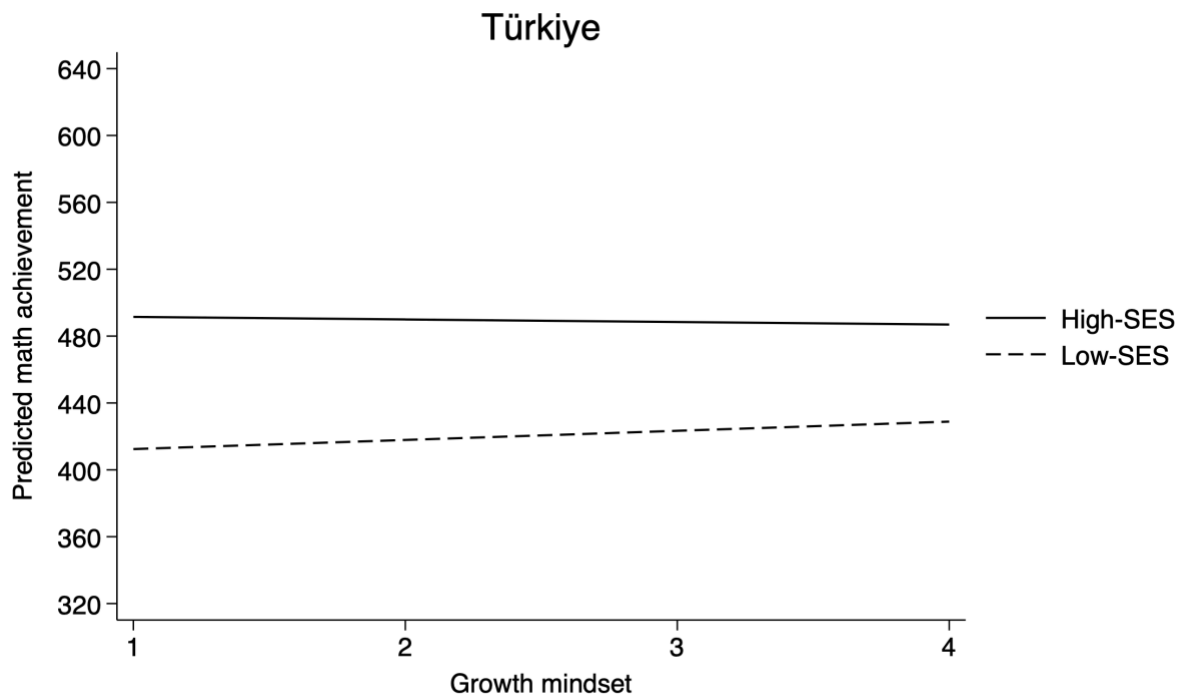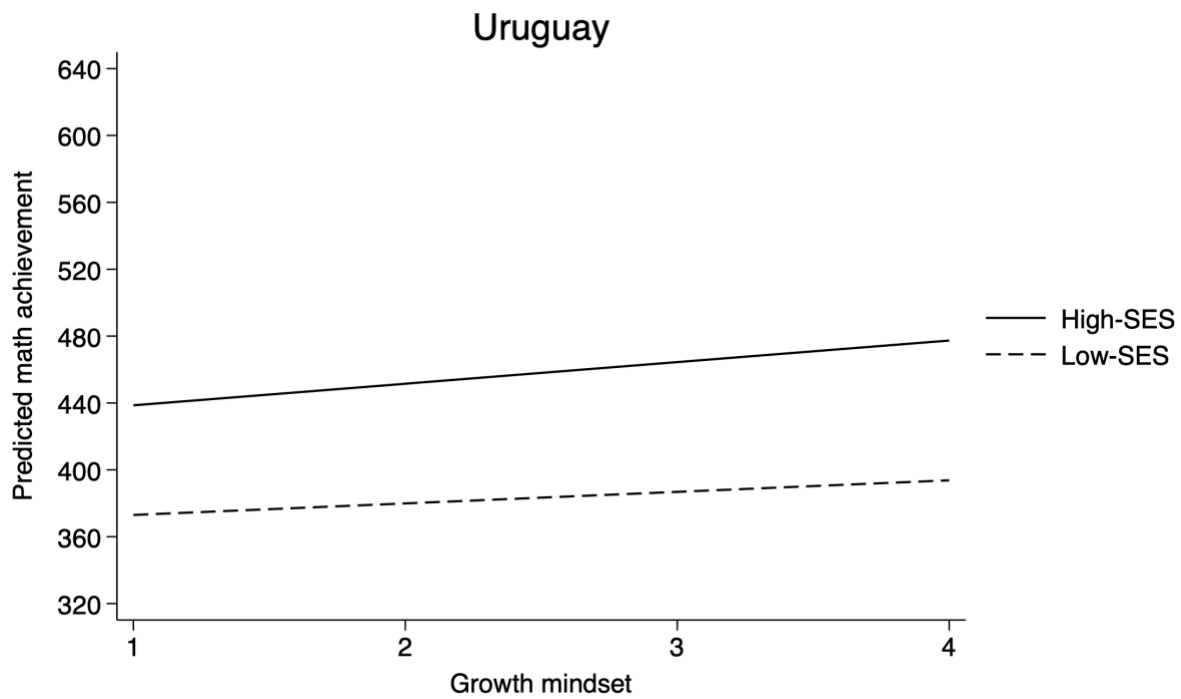

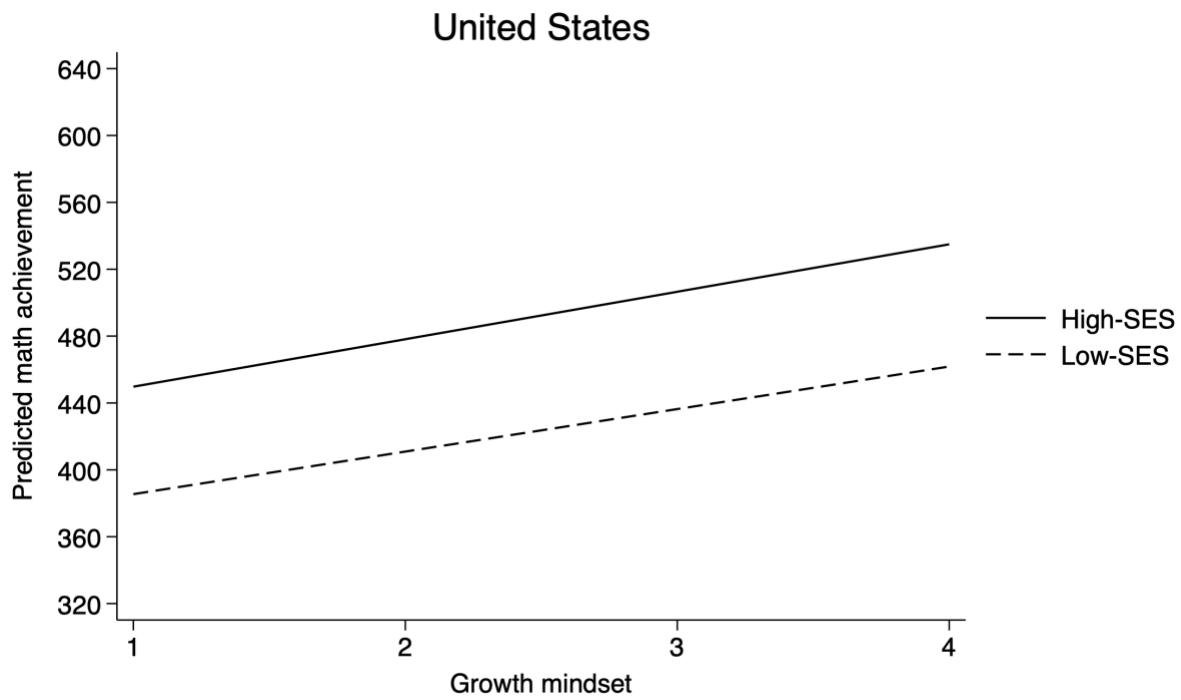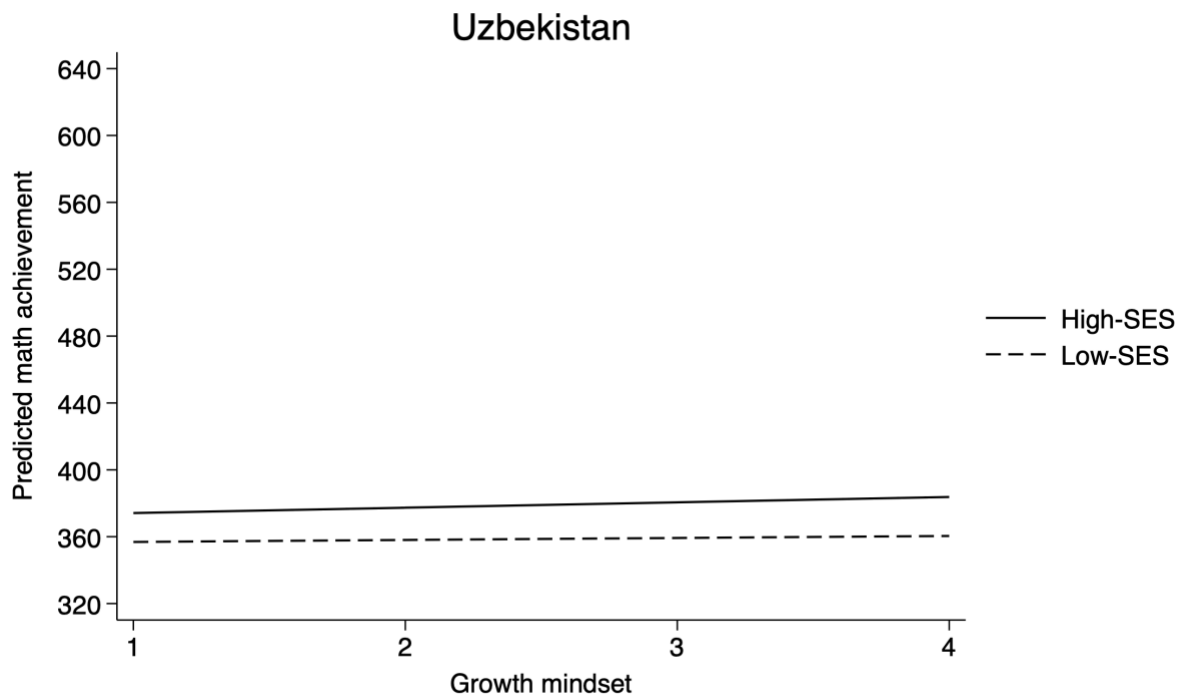

Supplement: S1 Appendix — (PDF) [file pone.0337039.s001.pdf]
