# Supplementary material for: Relationships between growth mindsets and math achievement across socioeconomic status in 74 countries: Evidence from PISA 2022
Source: PLoS One. 2025 Nov 21;20(11):e0337039. doi: 10.1371/journal.pone.0337039 (PMC12637945; doi:10.1371/journal.pone.0337039)
Supplement: S2 Appendix — (DOCX) [file pone.0337039.s002.docx]

**Robustness Analyses**

We tested three additional models to evaluate the robustness of the interaction between growth mindsets and socioeconomic status in predicting math achievement. Five additional variables retrieved from the public PISA 2022 dataset were employed as the covariates in the robustness testing models. The details of each variable are as follows:

**Gender** **(ST004D01T)**

Gender refers to the reported gender of a student, as validated by both school records and the students’ responses. In the original dataset, 1 represents female, while 2 refers to male. In the current study, the coding was transformed (Female = 1; Male = 0) to make female students the reference group compared to their male counterparts.

**Immigrant status (IMMIG)**

Immigrant status indicates a student’s immigrant background. Initially, 1 refers to native students; 2 refers to second-generation immigrant students, and 3 refers to first-generation immigrant students. To place immigrant students as the baseline, we recoded native students to 0, while both first- and second-generation immigrant students were coded as 1.

**Math anxiety (ANXMAT)**

According to OECD [1], the math anxiety score is scaled using the partial credit model (PCM), which incorporates six items that assess self-rated attitudes toward mathematics (e.g., “I feel anxious about failing in mathematics” and “I often worry that it will be difficult for me in mathematics classes”). The response options ranged from “Strongly agree”, “Agree”, “Disagree”, to “Strongly Disagree.”

**Math self-efficacy (MATHEFF)**

The math self-efficacy index captures students’ self-rated confidence about having to do nine formal and applied math questions (e.g., “Solving an equation like 2(x+3) = (x+3)(x-3)” and “Calculating how much more expensive a computer would be after adding tax”). The response options for these nine items included four points: “Not at all confident”, “Not very confident”, “Confident”, and “Very confident.” These items were scaled into the math self-efficacy construct using PCM.

**Math proactive behavior (MATHPERS)**

The math proactive behavior variable was scaled from eight items, which measure students’ self-rated frequency of how often they perform actions or behaviors that indicate effort and persistence in learning math (e.g., “I actively participated in group discussions during mathematics class” and “I put effort into my assignments for mathematics class”). Each item had five rating options, including “Never or almost never”, “Less than half of the time”, “About half of the time”, “More than half of the time”, and “All or almost all of the time.” This construct was scaled using a within-construct matrix sampling design [1].

After estimating the moderation model in predicting math achievement, we assessed the robustness of the interaction between growth mindsets and socioeconomic status by estimating three alternative model specifications. First, two demographic characteristics, gender and immigrant status, were added to the models. Gender is typically used to control for confounding variables when analyzing learning achievement using PISA data, given its potential influence on students’ academic outcomes [2,3]. At the same time, growth mindsets were often discussed in the contexts of students with disadvantaged or vulnerable backgrounds, particularly migrant students [4,5].

In the baseline model (Model 2), standardized coefficients ranged between -0.06 and 0.13 across countries. Adding two demographic controls, including students’ gender and immigrant status (Model 3), resulted in minor shrinkage of the standardized coefficients, which ranged from -0.05 to 0.12 (see S3 Table). Compared to the baseline specification, the changes were typically less than ±0.01, indicating that the effect of the interaction term remained robust when controlling for more complex demographic variables.

Thereafter, three additional non-cognitive abilities (math self-efficacy, math anxiety, and math proactive behaviors) were added to the second alternative model (Model 4). These non-cognitive variables were selected because recent studies have found both direct and indirect relationships between them and math achievement [6,7]. Incorporating these alternative non-cognitive factors tests the robustness of the interaction between growth mindsets and socioeconomic status when controlling for confounding self-rated variables.

When the alternative non-cognitive predictors were added, the range narrowed slightly (-0.05 to 0.09) as shown in Model 4 (see S4 Table). Changes in estimates were within ±0.01 to ±0.04 of the baseline model, except for the United Arab Emirates (-.06). The larger changes may reflect the theoretically and empirically supported mediation effects of growth mindset on these non-cognitive abilities when predicting academic achievements [8,9].

Lastly, the third specification (Model 5) incorporated a two-way interaction of growth mindsets with gender and immigrant status, while controlling for the three non-cognitive abilities from the previous specification (see S5 Table). The range of the standardized coefficient of the interaction between growth mindsets and socioeconomic status in Model 5 remained similar to that in Model 4. The changes in the estimates of Model 5 were typically within ±0.01 to ±0.04 of the baseline model in most countries. Substantive changes in coefficients (±0.06) were observed only in a few education systems, such as Qatar and the United Arab Emirates, where large baseline effects may amplify the interaction more noticeably. The summarized changes between each model compared to the baseline can be found in S6 Table. Overall, the robustness test results suggested that the moderating role of socioeconomic status in the association between growth mindsets and math achievement is robust across alternative specifications.

**References**

1. Organisation for Economic Co-operation and Development [OECD]. PISA 2022 technical report. Paris: OECD; 2024 Mar. doi:10.1787/01820d6d-en

2. Lee HJ, Mendoza NB. Does parental support amplify growth mindset predictions for student achievement and persistence? Cross-cultural findings from 76 countries/regions. Social Psychology of Education. 2025;28: 88. doi:10.1007/s11218-025-10038-4

3. Huang M, Liu X. Pathways to equity: A mediation analysis of gender, SES, and mathematics achievement using PISA 2022 UK data. Int J Educ Res. 2025;133: 102666. doi:10.1016/j.ijer.2025.102666

4. Kaya S, Eryilmaz N, Yuksel D. The effects of growth mindset and resilience on immigrant students’ PISA science achievement: The mediating role of attitudes toward school. Sage Open. 2024;14. doi:10.1177/21582440231225870

5. Boman B, Wiberg M. The influence of SES, migration background, and non-cognitive abilities on PISA reading and mathematics achievement: evidence from Sweden. European Journal of Psychology of Education. 2024;39: 2935–2951. doi:10.1007/s10212-024-00805-w

6. Shimizu Y. Relation between mathematics self-efficacy, mathematics anxiety, behavioural engagement, and mathematics achievement in Japan. Psychology International. 2025;7: 36. doi:10.3390/psycholint7020036

7. Zuo S, Huang Q, Qi C. The relationship between cognitive activation and mathematics achievement: mediating roles of self-efficacy and mathematics anxiety. Current Psychology. 2024;43: 30794–30805. doi:10.1007/s12144-024-06700-3

8. Huang X, Zhang J, Hudson L. Impact of math self-efficacy, math anxiety, and growth mindset on math and science career interest for middle school students: the gender moderating effect. European Journal of Psychology of Education. 2019;34: 621–640. doi:10.1007/s10212-018-0403-z

9. Samuel TS, Warner J. “I can math!”: Reducing math anxiety and increasing math self-efficacy using a mindfulness and growth mindset-based intervention in first-year students. Community Coll J Res Pract. 2021;45: 205–222. doi:10.1080/10668926.2019.1666063
