# Supplementary material for: Relationships between growth mindsets and math achievement across socioeconomic status in 74 countries: Evidence from PISA 2022
Source: PLoS One. 2025 Nov 21;20(11):e0337039. doi: 10.1371/journal.pone.0337039 (PMC12637945; doi:10.1371/journal.pone.0337039)
Supplement: Attachment — Submitted filename: Review_2.docx [file pone.0337039.s008.docx]

The requested corrections and additions have been made in the article. As a result, the findings are now linked to the empirical data and the existing theory, while also acknowledging limitations and proposing clear directions for future research.
In conclusion, it is now a well-prepared study and can be published..
